# RNAi-mediated knockdown of gut receptor-like genes prohibitin and α-amylase altered the susceptibility of *Galleria mellonella* to Cry1AcF toxin

**DOI:** 10.1186/s12864-022-08843-8

**Published:** 2022-08-18

**Authors:** Tushar K. Dutta, Abhishek Mandal, Artha Kundu, Victor Phani, Chetna Mathur, Arudhimath Veeresh, Rohini Sreevathsa

**Affiliations:** 1 Division of Nematology, ICAR-Indian Agricultural Research Institute, New Delhi, 110012 India; 2 Division of Agricultural Chemicals, ICAR-Indian Agricultural Research Institute, New Delhi, 110012 India; 3 Department of Agricultural Entomology, College of Agriculture, Uttar Banga Krishi Viswavidyalaya, Dakshin Dinajpur, Balurghat, West Bengal India; 4 ICAR-National Institute for Plant Biotechnology, New Delhi, 110012 India

**Keywords:** Prohibitin, GLTP, α-amylase, ADAM, UDP-GT, RNAi, Cry1AcF

## Abstract

**Background:**

Due to the prolonged usage of Bt-based biopesticides and Bt-transgenic crops worldwide, insects are continually developing resistance against Cry toxins. This resistance may occur if any mechanistic step in the insecticidal process is disrupted possibly because of the alteration in Cry-receptor binding affinity due to mutation in receptor genes. Compared to other lepidopteran insects, Cry receptor-related research has made asymmetric progress in the model insect *Galleria mellonella*.

**Results:**

Present study describes the molecular characterization and functional analysis of five Cry toxin receptor-related genes (prohibitin, GLTP, α-amylase, ADAM and UDP-GT) and a gut repair gene (arylphorin) from the gut tissues of *G. mellonella*. Protein–protein docking analysis revealed that Cry1AcF putatively binds with all the five candidate proteins, suggesting their receptor-like function. These receptor-like genes were significantly overexpressed in the gut tissues of fourth-instar *G. mellonella* larvae upon early exposure to a sub-lethal dose of Cry1AcF toxin. However, targeted knockdown (by using bacterially-expressed dsRNAs) of these genes led to variable effect on insect susceptibility to Cry1AcF toxin. Insects pre-treated with prohibitin and α-amylase dsRNA exhibited significant reduction in Cry1AcF-induced mortality, suggesting their probable role as Cry receptor. By contrast, insects pre-treated with GLTP, ADAM and UDP-GT dsRNA exhibited no significant decline in mortality. This maybe explained by the possibility of RNAi feedback regulation (as few of the receptors belong to multigene family) or redundant role of GLTP, ADAM and UDP-GT in Cry intoxication process.

**Conclusion:**

Since the laboratory culture of *G. mellonella* develop Bt resistance quite rapidly, findings of the current investigation may provide some useful information for future Cry receptor-related research in the model insect.

**Supplementary Information:**

The online version contains supplementary material available at 10.1186/s12864-022-08843-8.

## Background

Compared to other invertebrate models such as *Bombyx mori*, *Drosophila suzukii*, *D. melanogaster*, *Manduca sexta*, etc., the greater wax moth *Galleria mellonella* is a popular choice for investigating the insect immune responses towards alien microorganisms, virulent toxins or drug candidates [1]. A number of desirable features including the ease of culturing, greater number of offspring, shorter development cycle, translucent larval body and the availability of genome and immune-related transcriptome data has made *G. mellonella* an ideal model for deeper understanding of host immunity in vivo at biochemical and molecular levels [2]. Nevertheless, limited research advances have been made regarding the fate of *Bacillus thuringiensis* (Bt, an immensely important insect biocontrol agent)-secreted insecticidal Cry proteins in *G. mellonella* [3, 4]. Since the laboratory culture of *G. mellonella* develop Bt resistance quite rapidly compared to field populations of agriculturally-important insects [5], molecular and functional characterization of Bt receptor proteins putatively located in the gut tissues of *G. mellonella* would provide some valuable information towards our aim of unraveling the genetic basis of Bt resistance in insects.

Cry toxins are secreted as crystals from the sporulating Bt bacterium and upon delivery into the host insect gut lumen, Cry protoxin is activated by the enzymatic cleavage of gut proteases. Activated Cry traverses the peritrophic membrane and binds with a transmembrane receptor cadherin (CAD), located in the apical membrane of gut epithelial cell [6–9]. This causes a conformational change in toxin monomer to oligomer formation. Pre-pore oligomer then interacts with alkaline phosphatase (ALP) or alanyl aminopeptidase N (APN) receptors that are tethered in the lipid rafts of epithelial cell membrane via glycosylphosphatidylinositol (GPI) anchors. Upon insertion into the epithelial cell membrane, oligomer forms lytic pores and disrupts membrane integrity that eventually leads to insect death via septicemia [6–9]. Another pore formation model describes that Cry monomers first interact with APN followed by CAD for oligomer formation [10]. Additionally, a cell-signaling model proposes that binding of Cry monomers to CAD elicit a signal transduction pathway that ultimately causes cell death [11]. The involvement of the transmembrane receptor ABC transporter belonging to different subfamilies such as subfamily C (ABCC), subfamily A (ABCA), subfamily G (ABCG) and subfamily B (ABCB) in Cry intoxication process has also been demonstrated [12–14]. Taken together, the phenomena of toxin binding to various gut receptors is identical in all the models and is crucial for Cry mechanism of action because any alteration in the binding step may lead to Bt resistance development in insects [15].

Apart from CAD, ALP, APN and ABC transporters, a few receptor-like genes such as prohibitin, glycolipid transfer protein (GLTP), α-amylase, ADAM metalloprotease, uridine diphosphate-glucosyltransferase (UDP-GT) etc. have been shown to be involved in Cry intoxication process in different insects [7, 16–20]. In our earlier study, the independent and significant role of *G. mellonella* CAD, ALP, APN1 and ABCC2 receptors in Cry intoxication process was established [21]. In the present study, we interrogated the role of prohibitin, GLTP, α-amylase, ADAM and UDP-GT in Cry-induced toxicity in *G. mellonella*. We used a chimeric Cry toxin (protein-engineered toxins are used to confer broad-spectrum efficacy and delay the Bt resistance development in insects) Cry1AcF (by swapping the domains of Cry1Ac and Cry1F; patent number 237912) developed in our laboratory. Although these receptor-like genes were transcriptionally upregulated in midgut tissues of *G. mellonella* upon exposure to Cry toxin and exhibited binding affinity to Cry protein in in silico docking assay, the independent knockdown of these receptor genes caused variable alteration in insect susceptibility to Cry-induced toxicity. We included a candidate gut repair protein arylphorin in the analysis for comparison with the data of receptor-like genes.

## Results

### Identification of additional Cry toxin receptor-like genes from *G. mellonella*

A number of underexplored Cry receptor-like genes such as prohibitin, GLTP, α-amylase, ADAM, UDP-GT etc. and a gut repair gene, i.e. arylphorin (specifically overexpressed after Cry-induced damage) were characterized earlier from different insects [7, 16–20, 22]. To identify the putative homologues of these genes in the NCBI non-redundant database, we searched for the *G. mellonella* transcripts that hit these candidate genes using local BLASTn algorithm. We identified 1, 1, 1, 1, 5 and 49 unigenes corresponding to prohibitin, GLTP, α-amylase, ADAM, UDP-GT and arylphorin, respectively (*E*-value cut off < 1.0E^−30^). Either these homologous sequences belong to different regions of the identical gene or we assume them as allelic variants (Supplementary Table S1).

### Temporal expression profile of receptor-related genes in *G. mellonella* gut upon Cry1AcF delivery

**Fig. 1:**
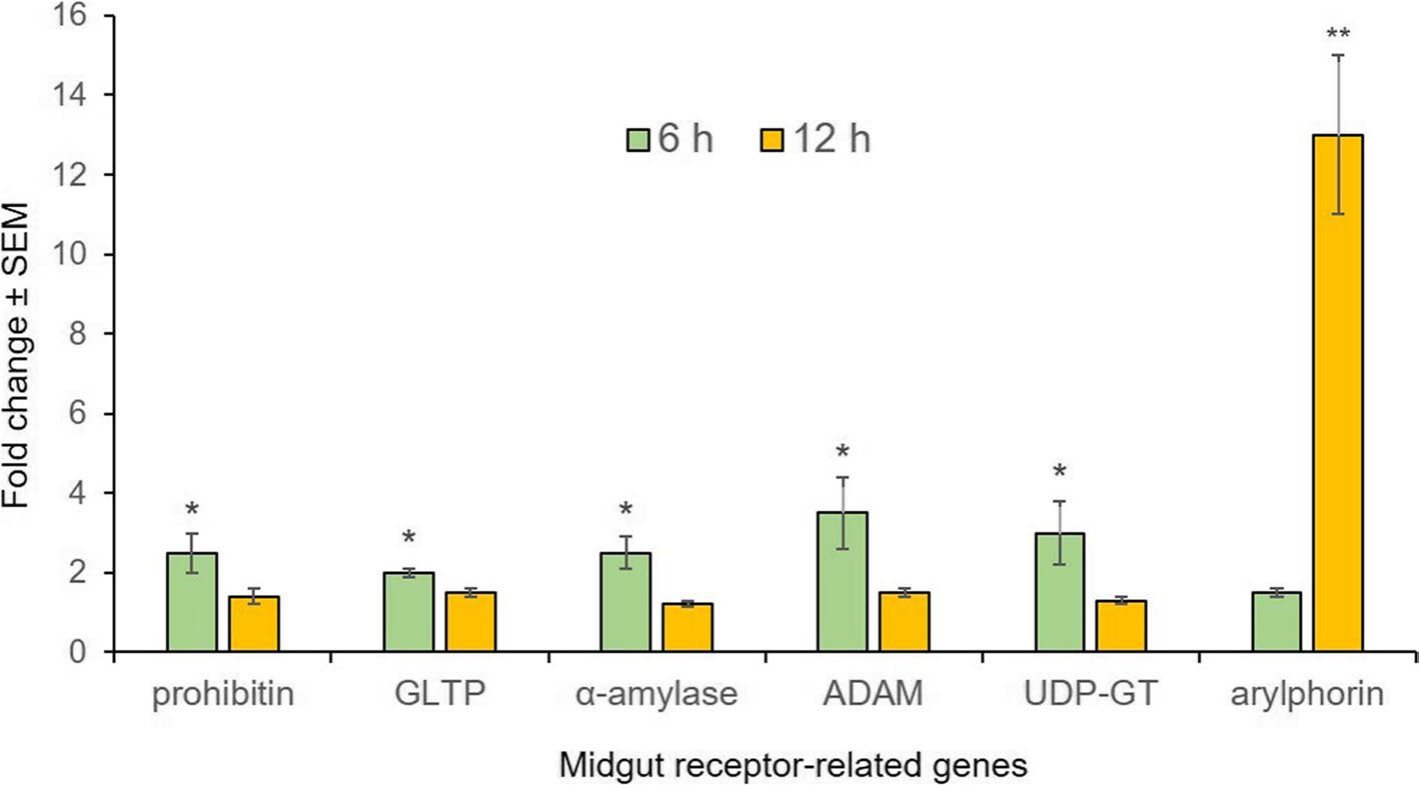
Differential expression of Cry receptor-like genes in the midgut of *G. mellonella* fourth-instar larvae at 6 and 12 h after oral administration of Cry1AcF toxin (dose: 27 ng per larva). Asterisk indicates significant (*P < 0.01, **P < 0.001; Tukey’s HSD test) difference in fold change value of the target gene compared to its baseline expression (fold change value set at 1) in control insect. *G. mellonella* housekeeping genes *18S rRNA* and *EF-1α* were used as internal reference. Each bar represents the mean fold change value ± SE of qPCR runs in five biological and three technical replicates

Fourth-instar *G. mellonella* larvae were force-fed with LD_50_ (27 ng/larva) dose (determined in our previous study; [19]) of Cry1AcF toxin and RNA was isolated from the midgut tissue at 6 h and 12 h after Cry ingestion, and RT-qPCR analysis was performed. A substantial overexpression of prohibitin, GLTP, α-amylase, ADAM and UDP-GT (*P* < 0.01) mRNAs was observed at 6 h after Cry inoculation, compared to PBS (control)-treated larvae (Fig. 1). On the contrary, expression of arylphorin was not differentially altered (*P* > 0.01) in the Cry1AcF-treated larvae compared to control at that time point (Fig. 1). Nevertheless, at 12 h after Cry inoculation, prohibitin, GLTP, α-amylase, ADAM and UDP-GT transcripts were not differentially expressed (*P* > 0.01) in the toxin-treated larvae compared to control. Intriguingly, arylphorin mRNAs were greatly upregulated (*P* < 0.01) in the toxin-treated insects compared to control at 12 h time point (Fig. 1). This is indicative of initiation of gut repair process in the midgut of toxin-treated insects.

### Cry1AcF binds with different receptor proteins in in silico docking assay

**Fig. 2:**
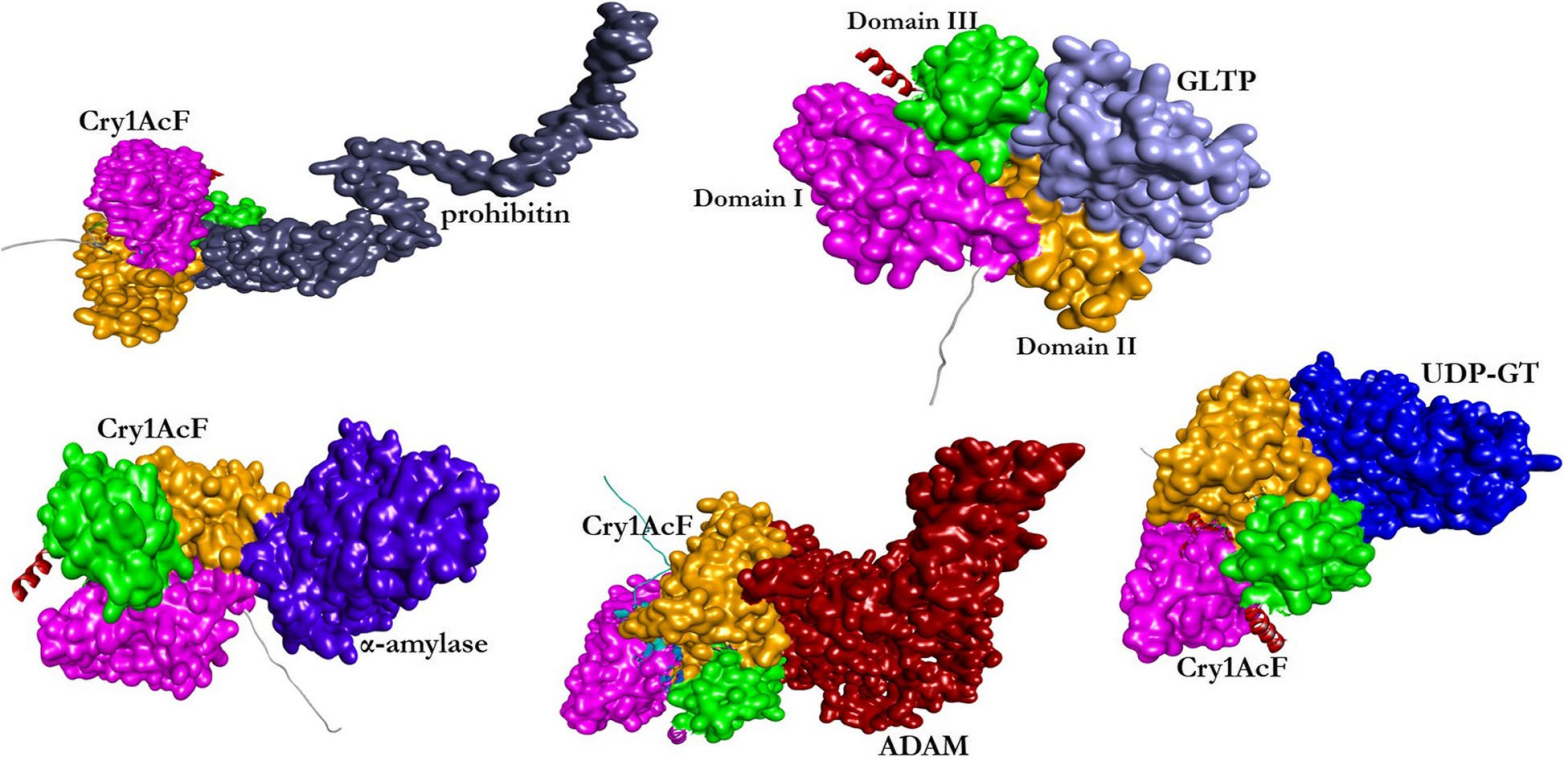
Protein–protein interaction between Cry1AcF ligand and putative gut receptors prohibitin, GLTP, α-amylase, ADAM and UDP-GT. Cry1AcF bound with these receptors via a number of Pi interactions, hydrogen bonds and salt bridges. The ZDock scores (greater value indicates greater contact surface area between ligand and receptor) for Cry-prohibitin, Cry-GLTP, Cry-α-amylase, Cry-ADAM and Cry-UDP-GT complexes were 1510, 1961, 2041, 1916 and 1836 Å^2^, respectively. Domain I, II and III of Cry1AcF are highlighted in magenta, ochre yellow and green color, respectively. Exposed α helices and β sheets (with default colors) do not encompass any domain of Cry1AcF

The chimeric toxin Cry1AcF was generated by swapping the domains of Cry1Ac (Domain I and II) and Cry1F (Domain III) toxin ([23]; patent number 237912). Cry1AcF Domain I (N-terminal) is associated with membrane insertion and pore formation, i.e. catalytic activity; Domain II helps in receptor binding; Domain III (C-terminal) perform several functions including acting as a major determinant of receptor binding [24]. Initially, using homology modelling best templates were selected for the ligand and receptors. Protein–protein docking analysis revealed that Cry1AcF bound with these receptors via a number of Pi interactions, hydrogen bonds and salt bridges. The ZDock scores (include contact surface area of both polar and non-polar amino acid residues; greater value indicates greater contact surface area between ligand and receptor) for Cry-prohibitin, Cry-GLTP, Cry-α-amylase, Cry-ADAM and Cry-UDP-GT complexes were 1510.855, 1961.207, 2041.419, 1916.326 and 1836.010 Å^2^, respectively (Fig. 2; Supplementary Table S2). Expectedly, all these receptors interacted mostly with the Domain II and Domain III of Cry1AcF ligand (Fig. 2). In addition, Cry1AcF was docked with its known receptors including CAD, ABCC2, ALP and APN1 as reference. The ZDock scores for Cry-CAD, Cry-ABCC2, Cry-ALP and Cry-APN1 complexes were 2321, 1907, 2189 and 2060 Å^2^, respectively (Supplementary Figure S1; Supplementary Table S2). The comparable ZDock scores are indicative of validity of the docking models in establishing the putative role of prohibitin, GLTP, α-amylase, ADAM and UDP-GT as receptors for Cry proteins.

### Molecular characterization of receptor-related genes from *G. mellonella*

By employing 3´, 5´ RACE-PCR and primer walking, the coding sequences (RNA was extracted from *G. mellonella* fourth-instar midgut tissue) of prohibitin, GLTP, α-amylase, ADAM, UDP-GT and arylphorin were obtained. NCBI Genbank accession numbers obtained for these sequences are provided in Table 1. Information regarding the open reading frame (ORF), encoded amino acid (aa) numbers, obtained NCBI Genbank accession numbers, predicted molecular weight and isoelectric point (pI, lower value is indicative of greater protein stability) of these candidate receptors are listed in Table 1.

**Table 1:** Basic information of different Bt receptor-like genes, a gut repair gene and their proteins in *G. mellonella*

Gene	Locus ID	Gene length (bp)	Number of Exons	CDS length (bp)	^a^Accession Number	Predicted Protein		
						Size (aa)	MW (kDa)	pI
prohibitin	LOC113512717	3609	5	1168	MZ561435	274	30.00	6.35
GLTP	LOC113517303	3974	5	1776	MZ576277	208	24.11	7.68
α-amylase	LOC113522752	100802	11	1869	MZ576276	623	68.53	7.56
ADAM	LOC113515341	51805	17	4476	MZ576274	990	111.99	8.12
UDP-GT	LOC113510033	4642	4	1852	MZ561436	431	49.67	6.93
arylphorin	LOC113516268	3829	5	2195	MZ576275	702	83.37	5.09

^a^Coding sequences with accession numbers are cloned and sequenced from the midgut mRNA sample of *G. mellonella* fourth instar larvae in our laboratory and are available in the NCBI database (https://www.ncbi.nlm.nih.gov/nucleotide/)

**Fig. 3:**
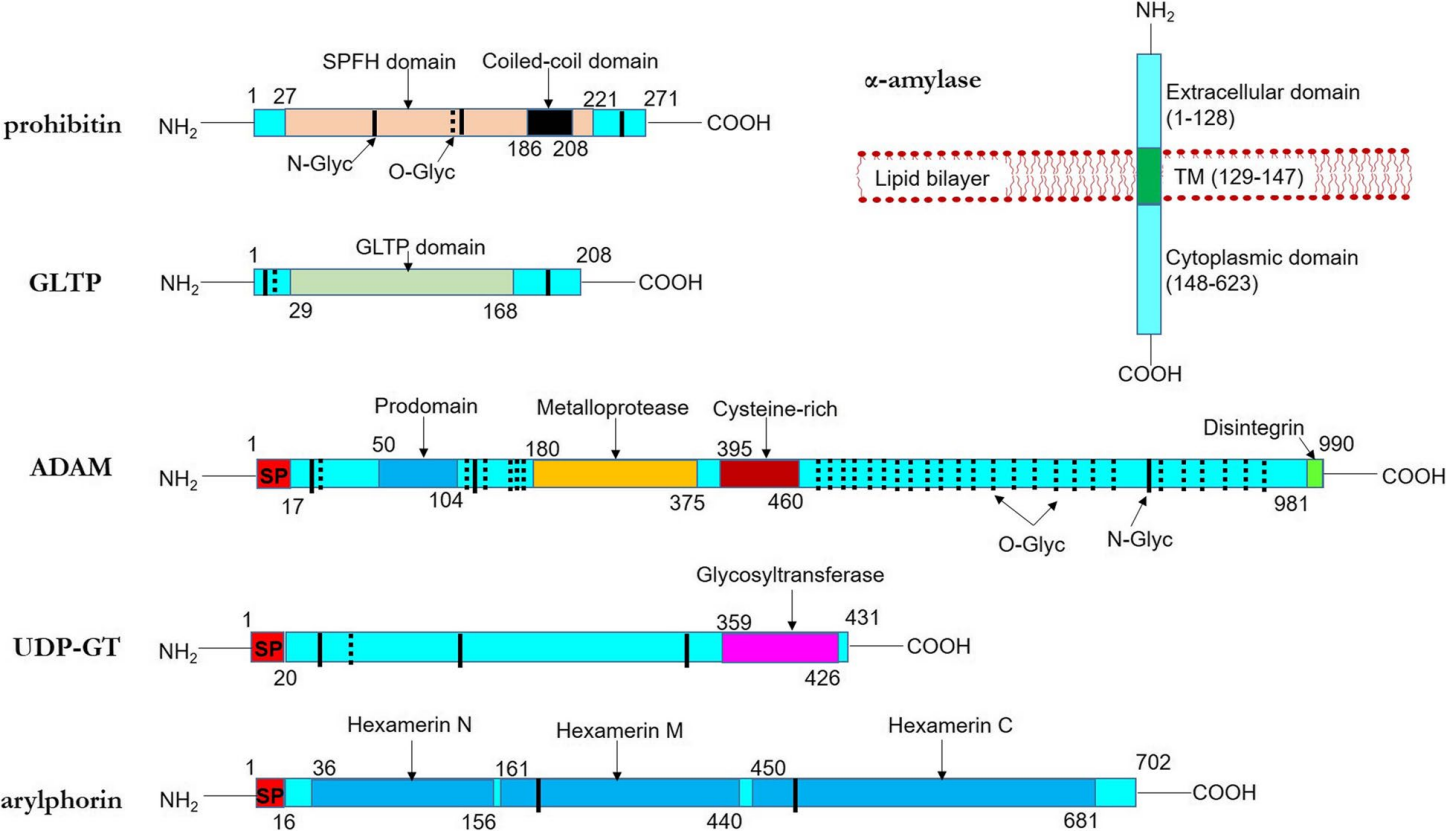
Predicted secondary structures of five Cry receptor-like proteins and a gut repair protein. Membrane anchored prohibitin contains SPFH (wheatish box) and coiled-coil (black box) domains. Intracellular GLTP contains a characteristic GLTP domain (light green box). α-amylase contains an intracellular, extracellular and a transmembrane (dark green box) domain. Extracellular ADAM includes N-terminal signal peptide, a prodomain (blue box), metalloprotease catalytic site (yellow box), cysteine-rich region (red box), C-terminal disintegrin domain (green box) and numerous N- and O-glycosylated residues. Extracellular UDP-GT includes N-terminal signal peptide, C-terminal UGT signature motif (magenta box) and few N/ O-glycosylated residues. Extracellular arylphorin includes a signal peptide and three Hexamerin domains (blue boxes). Solid and dotted perpendicular lines indicate N- and O-glycosylated residues, respectively

The predicted prohibitin (274 aa) contains a membrane located SPFH domain (27–221 aa), a coiled-coil domain (186–208 aa) and a few N-glycosylated asparagine and O-glycosylated serine/threonine residues (Fig. 3). GLTP (208 aa) is a cytosolic protein that putatively function in intermembrane lipid transfer. α-amylase is a transmembrane protein with putative protein binding activity (1–128 aa: extracellular; 129–147 aa: membrane spanning; 148-623aa: cytosolic). The extracellular ADAM (990 aa) consists of a N-terminal signal peptide, a propeptide domain (50–104 aa), a metalloprotease domain containing the conserved HEXXHXXGXXH motif (180–375 aa), a cysteine-rich region (395–460 aa), a C-terminal disintegrin domain containing RGD motif (981–990 aa), 9 N-glycosylated and 41 O-glycosylated residues (Fig. 3). The extracellular UDP-GT (431 aa) contains a N-terminal signal peptide, a C-terminal glycosyltransferase signature domain (359–426 aa), and a few N- and O-glycosylated residues (Fig. 3). The extracellular arylphorin (702 aa) consists of a N-terminal signal peptide, a number of hexamerin (larval storage protein) signature motifs (RDP, GFP) and a few N-glycosylated residues (Fig. 3).

Interestingly, ADAM, prohibitin, GLTP and arylphorin protein of *G. melonella* had homologous sequences in different insect orders. Phylogenetic analysis showed that ADAM, prohibitin, GLTP and arylphorin sequences are evolutionarily highly conserved in their corresponding insect orders as lepidopteran clades branched away from coleopteran, isopteran, hymenopteran, hemipteran, dipteran, dictyopteran clades etc. (Supplementary Figures S2-S5). However, UDP-GT and α-amylase had no homologues in insect orders other than Lepidoptera (Supplementary Figures S6-S7). ADAM and prohibitin motif numbers and their location were highly conserved across the different insect orders (Supplementary Figure S8-S9), suggesting the functional similarities of ADAM and prohibitin in different insect orders. Similar trend was observed with UDP-GT, α-amylase and arylphorin (Supplementary Figures S10-S12). By contrast, motif characteristics of GLTP varied considerably (although motifs were conserved within the lepidopteran members) across the different insect orders (Supplementary Figure S13), suggesting the functional divergence of GLTP protein in insects.

### Stage- and tissue-specific expression profiles of receptor-related genes in *G. mellonella*

**Fig. 4:**
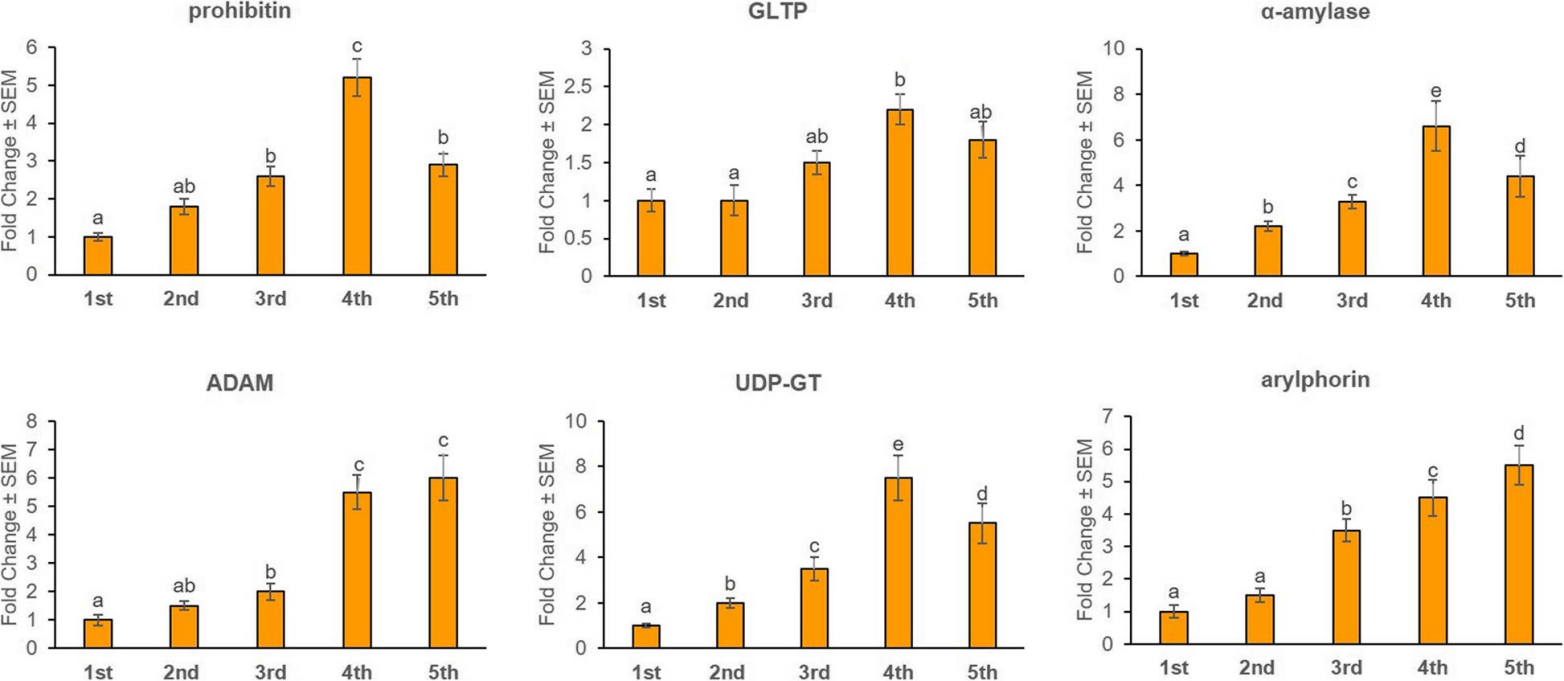
Stage-specific expression patterns of Cry receptor-related genes and a gut repair gene in different life stages of *G. mellonella*. Fold change in expression of a candidate gene in different developmental (second-, third-, fourth- and fifth-instar) stages was quantified in relation to the gene’s expression in first-instar stage (value set at 1). Significant differential expression is indicated by different letters (*P* < 0.01, Tukey’s HSD test). *G. mellonella 18S rRNA* and *EF-1α* genes were used as the internal reference. Each bar represents the mean fold change value ± SE of qPCR runs in five biological and three technical replicates

Transcription profile of candidate receptor mRNAs was analyzed by qPCR in different developmental stages of *G. mellonella* and in different body parts of *G. mellonella* fourth-instar stage. Transcripts of prohibitin, α-amylase and UDP-GT were most abundantly expressed (*P* < 0.01) in the fourth-instar stage compared to other life stages (Fig. 4). ADAM was greatly expressed in both fourth- and fifth-instar stages; GLTP expression did not significantly differ (*P* > 0.01) in third-, fourth- and fifth-instar stages; arylphorin was most abundantly expressed in the fifth-instar stage (Fig. 4).

**Fig. 5:**
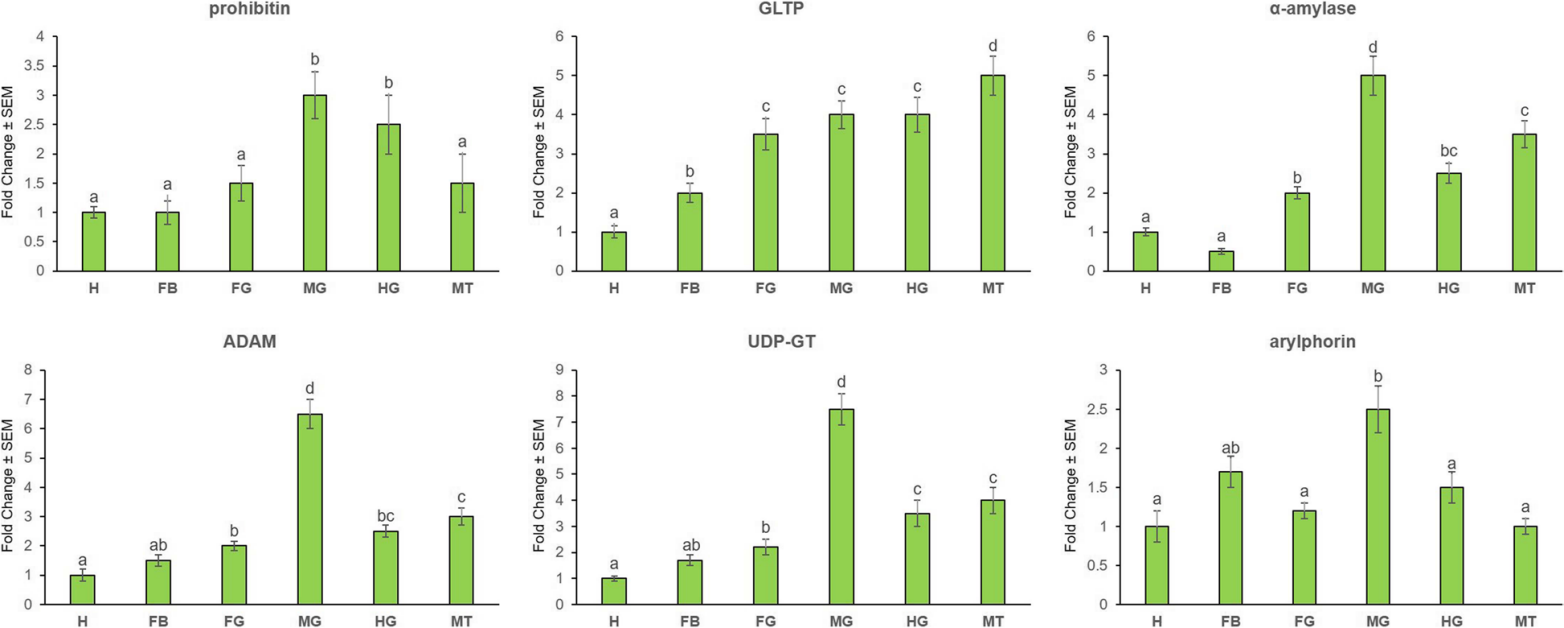
Tissue-specific expression patterns of Cry receptor-related genes and a gut repair gene in different tissues of *G. mellonella* fourth-instar larvae. Fold change in expression of a candidate gene in different tissues (fat body (FB), foregut (FG), midgut (MG), hindgut (HG) and Malpighian tubules (MT)) was quantified in relation to the gene’s expression in head (H) tissue (value set at 1). Significant differential expression is indicated by different letters (*P* < 0.01, Tukey’s HSD test). *G. mellonella 18S rRNA* and *EF-1α* genes were used as the internal reference. Each bar represents the mean fold change value ± SE of qPCR runs in five biological and three technical replicates

α-amylase, ADAM and UDP-GT mRNAs were highly upregulated (*P* < 0.01) in the midgut tissues compared to other body parts such as head, fat body, foregut, hindgut and Malpighian tubules (Fig. 5). However, prohibitin expression did not significantly differ (*P* > 0.01) in midgut and hindgut tissues; greatest expression (*P* < 0.01) of GLTP was observed in Malpighian tubules; arylphorin expression did not significantly differ (*P* > 0.01) in midgut and fat body tissues (Fig. 5). Collectively, majority of the Cry-toxin receptor-related genes were greatly expressed in the fourth-instar larval stages and midgut tissues of *G. mellonella*.

## RNAi of receptor-related genes conferred variable effect on *G. mellonella* susceptibility to Cry1AcF

Target dsRNA molecules were designed from the coding sequences of prohibitin (387 bp), GLTP (344 bp), α-amylase (390 bp), ADAM (365 bp), UDP-GT (307 bp) and arylphorin (382 bp), by adopting the following predetermined criteria: (i) target size should be less than 400 bp for efficient processing of dsRNA molecule by RNaseIII nuclease or Dicer, (ii) targeted region must predict greater siRNA generation probabilities than the untargeted region. In addition, target dsRNA sequences were used as the query against the *Drosophila melanogaster* siRNA database in order to investigate the probability of RNAi-induced off-target effects. DsCheck (http://dscheck.rnai.jp/) output indicated the negligible homology of *Drosophila* siRNAs with that of siRNAs formed from dsRNAs of prohibitin, GLTP, α-amylase, ADAM, UDP-GT and arylphorin (data not shown). Since dsRNA-expressing bacteria can synthesize intact dsRNA molecules in the larval gut, *E. coli* HT115 cells (RNase III deficient) harboring the recombinant L4440 containing dsRNAs (yield of bacterially-expressed dsRNA was approximately 10 μg in 20 μL PBS) corresponding to different receptor-related genes were independently force-fed into *G. mellonella* fourth-instar larvae using a 26 gauge hypodermic syringe. As control, insects were ingested with PBS. To assess whether dsRNA itself has any negative effect on insects, *G. mellonella* larvae were separately ingested with GFP dsRNA as a non-native control.

**Fig. 6:**
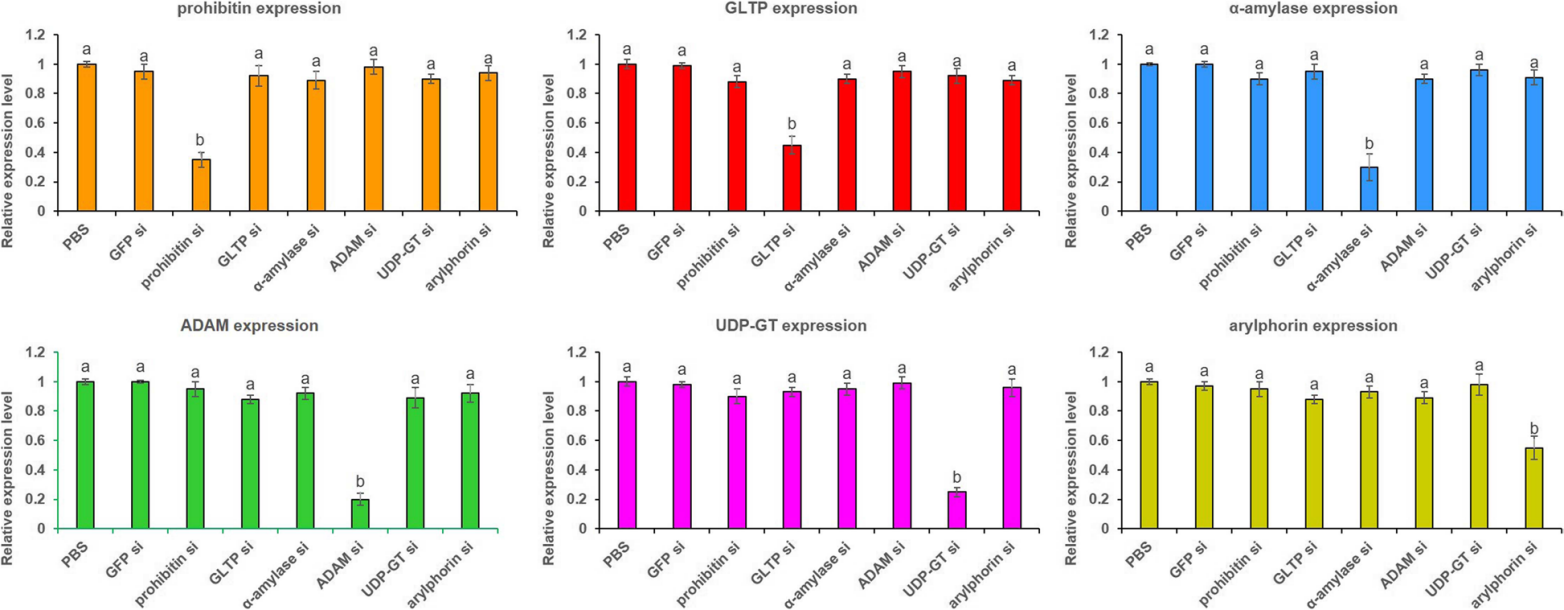
RNAi knockdown of five receptor-related genes and a gut repair gene in *G. mellonella* midgut led to target-specific silencing. Abundance of transcripts corresponding to prohibitin, GLTP, α-amylase, ADAM, UDP-GT and arylphorin in insects silenced (si) with these target genes were analyzed by qPCR. Fourth-instar larvae were force-fed with dsRNA-expressing *E. coli* HT115 cells and inoculated for 24 h. Insects orally ingested with GFP dsRNA and PBS were used as the non-native and negative control, respectively. *G. mellonella 18S rRNA* and *EF-1α* genes were used as the internal reference. Each bar represents the mean fold change value ± SE of qPCR runs in five biological and three technical replicates. Significant differential expression is indicated by different letters (*P* < 0.01, Tukey’s HSD test)

**Fig. 7:**
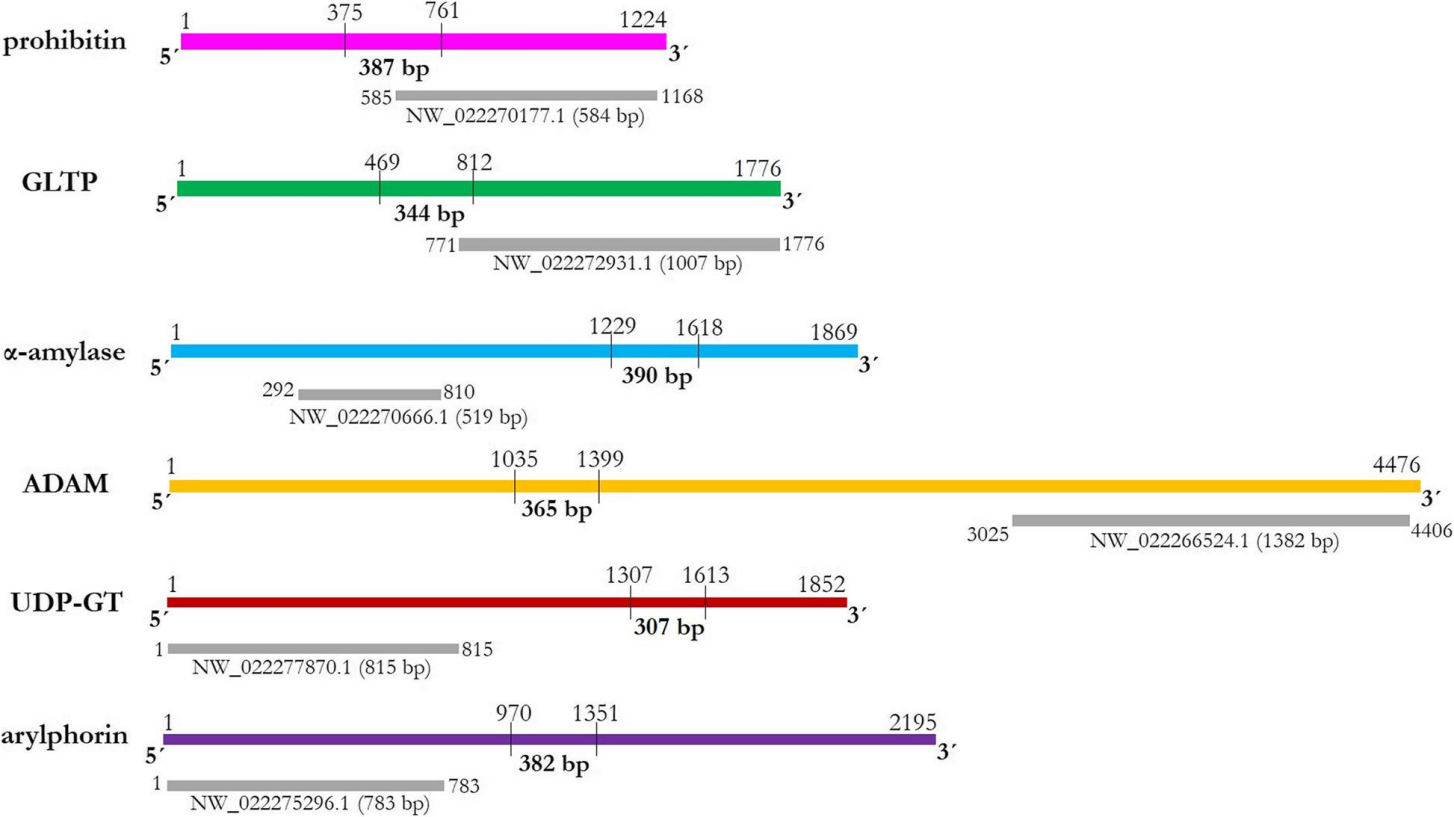
The dsRNA binding sites (indicated by perpendicular lines) in the coding sequences of *G. mellonella* prohibitin, GLTP, α-amylase, ADAM, UDP-GT and arylphorin are shown. Additionally, the homologous transcripts (indicated by grey boxes) that were aligned with the target receptors are shown. Numbers indicate the sequence coordinates

Prohibitin, GLTP, α-amylase, ADAM, UDP-GT and arylphorin mRNAs in the larval gut were significantly (*P* < 0.01) reduced by 65, 55, 70, 80, 75 and 45% in prohibitin, GLTP, α-amylase, ADAM, UDP-GT and arylphorin dsRNA-treated insects, respectively, compared to PBS at 24 h (Fig. 6). GFP dsRNA treatment did not alter (*P* > 0.01) the transcription of either of the receptor-related genes (Fig. 6). Transcript abundance of prohibitin was unaltered (*P* > 0.01) in GLTP, α-amylase, ADAM, UDP-GT and arylphorin dsRNA-treated insects. Vice versa was documented with GLTP, α-amylase, ADAM, UDP-GT and arylphorin expression (Fig. 6). This exemplifies the target-specific silencing of Cry receptor-related genes via RNAi. This may further be supported by the fact that largest homologous transcripts of α-amylase (NW_022270666.1), ADAM (NW_022266524.1), UDP-GT (NW_022277870.1) and arylphorin (NW_022275296.1) did not contain the dsRNA-binding regions, which we targeted for the present study (Fig. 7). Homologous transcripts of prohibitin (NW_022270177.1) and GLTP (NW_022272931.1) partially contained the dsRNA-binding site (Fig. 7).

**Fig. 8:**
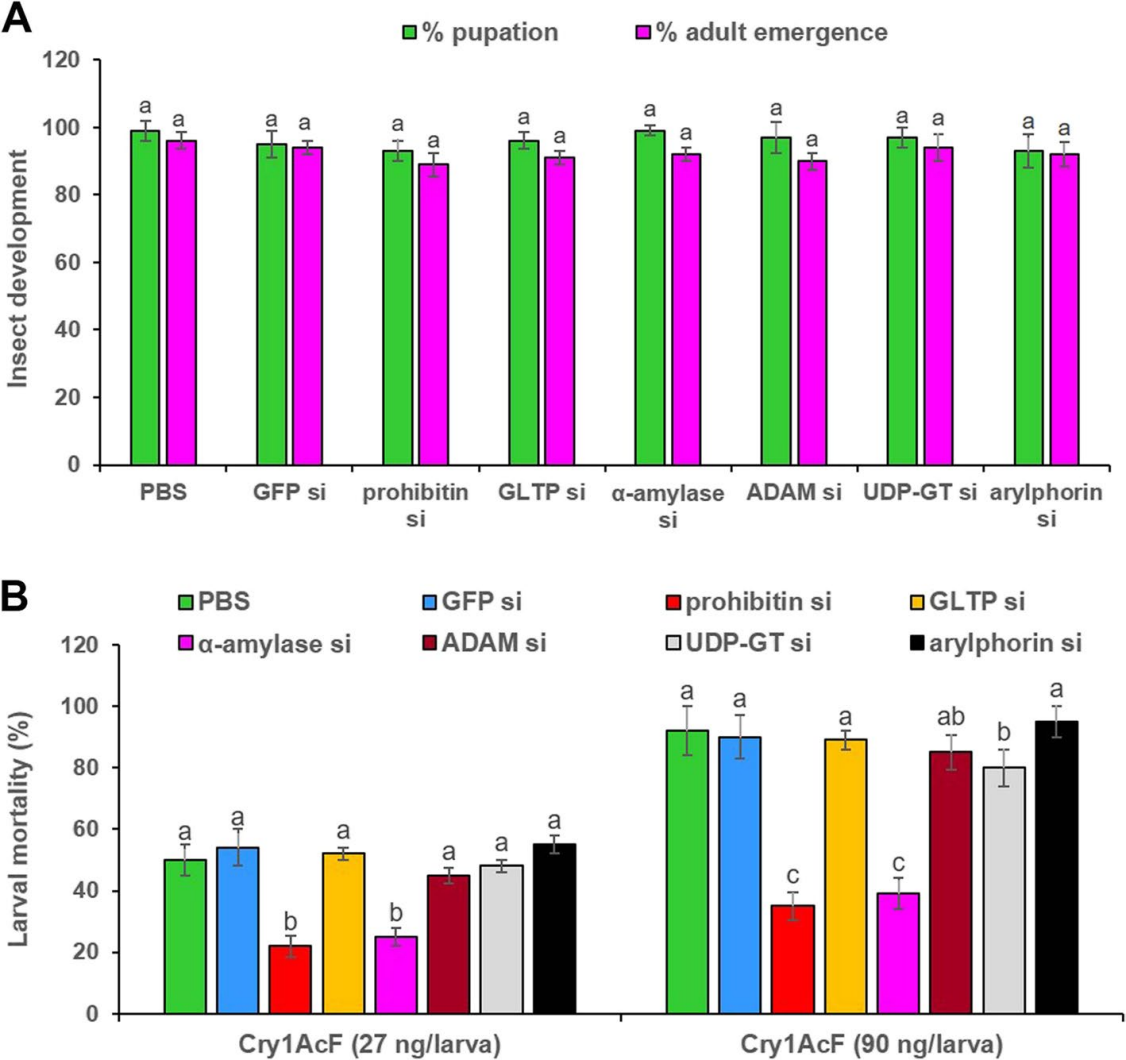
RNAi-induced downregulation of Cry receptor-related genes differentially altered the insect susceptibility to Cry1AcF toxin. **A** Percent pupation and adult emergence data of dsRNA-treated *G. mellonella* during 7 to 10 days after ingestion. Each bar with same letter is indicative of no significant difference between treatments (*P* > 0.01, Tukey’s HSD test, *n* = 30). **B** Percent mortality data of Cry1AcF force-fed larvae, which were pre-inoculated with dsRNAs corresponding to five Cry receptor-related genes and one gut repair gene. Post 24 h of dsRNA treatment, LD_50_ (27 ng per larva) and LD_90_ (90 ng per larva) doses of Cry1AcF were orally delivered. After another 24 h mortality data was recorded. Different letters are indicate significant difference between treatments (*P* < 0.01, Tukey’s HSD test, *n* = 50)

RNAi-induced downregulation of prohibitin, GLTP, α-amylase, ADAM, UDP-GT and arylphorin transcripts in larval gut did not cause any negative effect on insect growth and development as assessed by the percent pupation and adult emergence data, observed at 7 to 10 days after dsRNA inoculation (Fig. 8a).

At 24 h post dsRNA treatment, larvae were orally ingested with Cry1AcF toxin in sub-lethal (LD_50_: 27 ng per larva) and lethal (LD_90_: 90 ng per larva) doses (determined in our earlier study; [19]). At 24 h after toxin administration and incubation in artificial diet, insect mortality data were obtained. Compared to PBS, Cry1AcF-induced (27 ng/larva) mortality in prohibitin and α-amylase dsRNA pretreated insects were reduced (*P* < 0.01) by 56 and 50%, respectively. At lethal dose (90 ng/larva), Cry1AcF-induced mortality in prohibitin and α-amylase dsRNA pretreated larvae reduced (*P* < 0.01) by 61.95 and 57.61%, respectively, compared to PBS (Fig. 8b). This establishes the role of prohibitin and α-amylase as potential receptors for Cry intoxication in *G. mellonella* as induced knockdown of these genes reduced the insect sensitivity to Cry1AcF toxin. However, induced knockdown of GLTP and ADAM did not significantly (*P* > 0.01) alter the *G. mellonella* susceptibility to Cry1AcF toxin at both lethal and sub-lethal doses (Fig. 8b). RNAi of UDP-GT reduced larval mortality by a meagre 13% (*P* < 0.01) in Cry1AcF-treated (lethal dose) insects, compared to control; although at sub-lethal dose no significant change (*P* > 0.01) with control was observed (Fig. 8b). No significant difference (*P* > 0.01) in insect mortality data between arylphorin silenced and control insects (Fig. 8b) is expected because arylphorin is not a Cry receptor protein.

## Discussion

Current report describes the molecular characterization and functional analysis of five Cry toxin receptor-related genes (prohibitin, GLTP, α-amylase, ADAM and UDP-GT) and a gut repair gene (arylphorin) from the gut tissues of an insect model *G. mellonella*. Our protein–protein docking analysis revealed that Cry1AcF putatively binds with all the five candidate proteins, suggesting their receptor-like function. Additionally, a number of N- and O-Glycosylated residues were also predicted in the amino acid sequences of *G. mellonella* prohibitin, GLTP, α-amylase, ADAM and UDP-GT. Glycoproteins such as N- and O-glycans can covalently bind to proteins and lipids and form glycoconjugates. Glycoconjugates such as N-acetylgalactoseamine (GalNAc) tether to the cell membrane lipids via glycosylphosphatidylinositol (GPI) anchors that are embedded in the membrane. Notably, TcdA1 toxin isolated from *Photorhabdus luminescens* bacterium bound to the N-glycans in insect and mammalian cell lines, and GalNAc-deficient cell lines averted TcdA1-induced toxicity [25]. Similarly, the known receptors of Cry toxin such as CAD, ABCC2, ALP and APN1 contain a number of N- and O-Glycosylated residues. The interaction of activated Cry toxin with membrane-bound glycosylated receptors lead to Cry oligomer formation, an essential step in Cry mode of action [6, 26].

ADAM protein (a zinc-binding metalloprotease) characterized in Coleopteran beetles is a transmembrane receptor that binds with Cry3Aa toxin [27]. However, our reported ORF of ADAM from *G. mellonella* did not predict any transmembrane domain although other ADAM-specific signature domains such as propeptide, metalloprotease, cysteine-rich and disintegrin were predicted. *G. mellonella* ADAM homologues. Presence of a reduced complement of ADAM protein in *G. mellonella* maybe explained by the possibility of functional redundancy of ADAM receptor for Cry intoxication as a number other glycosylated proteins function as Cry receptor in *G. mellonella*. Our phylogenetic analyses showed that ADAM sequences are evolutionarily highly conserved in their corresponding insect orders as lepidopteran clade branched away from coleopteran, isopteran and hymenopteran clades. UDP-GT proteins putatively function as detoxifying enzyme in multi-insecticide resistance. UDP-GTs identified in *Plutella xylostella* contains a C-terminal transmembrane domain [28]. Nevertheless, our reported ORF of UDP-GT from *G. mellonella* did not predict any transmembrane domain although the signature glycosyltransferase domain was predicted. Insect-specific functional redundancy of UDP-GT is assumed here considering that UDP-GT is a multigene family and their homologues have been found in different insect orders [29]. According to our phylogeny analysis, *G. mellonella* UDP-GT homologues are exclusively present in order Lepidoptera.

A GPI-anchored α-amylase protein was identified in a dipteran pest, *Anopheles albimanus*, as the novel receptor for Cry4Ba and Cry11Aa [16]. Although the α-amylase we report from *G. mellonella* does not contain the GPI anchor, but is a transmembrane protein that contains both extracellular domain and a cytoplasmic tail. Surprisingly, *G. mellonella* α-amylase homologues were not found outside the order Lepidoptera. On the contrary, *G. mellonella* prohibitin homologues were detected in diverse insect species belonging to 10 orders. Prohibitin belongs to SPFH protein family (lipid raft proteins) and putatively anchors with the plasma membrane via interaction with the actin cytoskeleton. Prohibitin was demonstrated as the receptor (for Cry3Aa toxin) in Colorado potato beetle’s larval midgut membrane [18]. Prohibitin motif characteristics are highly conserved across the different insect orders suggesting their functional similarity. Conversely, motif characteristics of GLTP (in *G. mellonella*, cytosolic localization predicted) protein varied greatly in different insect orders implying their functional diversity. Arylphorin (identified in the secretome of *Heliothis virescens* midgut cell culture) putatively functions in the midgut regeneration process after midgut degeneration due to Cry1Ac intoxication [22]. Notably, our RT-qPCR analysis indicated a 13-fold upregulation of arylphorin mRNAs in the larval midgut of *G. mellonella* at 12 h after Cry1AcF oral ingestion, compared to control.

In order to examine the physiological roles of Cry receptor-related genes in *G. mellonella*, the expression profiles of prohibitin, GLTP, α-amylase, ADAM and UDP-GT mRNA were analyzed by RT-qPCR in different life stages and body parts of *G. mellonella*. With few exceptions majority of these candidate genes were predominantly transcribed in the midgut tissues and fourth-instar larval stages of *G. mellonella*. This is in agreement with our previous finding that *G. mellonella* fourth-instar stage is most vulnerable to Cry1AcF-induced toxicity and Cry1AcF enzymatic action occurs in midgut epithelium as revealed by the histopathology assay [21]. The non-receptor gene arylphorin was greatly expressed in the fifth-instar stage of *G. mellonella* in the current study.

In order to interrogate the decisive role of these receptor-related genes in Cry1AcF-induced toxicity, independent RNAi knockdown of prohibitin, GLTP, α-amylase, ADAM and UDP-GT was carried out in *G. mellonella* fourth-instar larvae. Target-specific silencing of the candidate receptors in midgut tissue was documented because oral delivery of bacterially-expressed dsRNAs corresponding to prohibitin, GLTP, α-amylase, ADAM and UDP-GT reduced the expression of prohibitin, GLTP, α-amylase, ADAM and UDP-GT genes in the gut tissue by 65, 55, 70, 80 and 75%, respectively, compared to control. Induced silencing of an independent receptor did not significantly alter the expression of other receptors in the current study, eliminating the possibility of any cross-silencing effect. Notably, only 25% discontinuous sequence conservation (with absence of any short stretches of homologous sequences) was detected among the targeted dsRNA sequences of prohibitin, GLTP, α-amylase, ADAM and UDP-GT (data not shown). In addition, silencing of the receptor-related genes did not confer any negative effect on *G. mellonella* growth and development. However, induced downregulation of the target genes caused variable effect on insect susceptibility to Cry1AcF toxin. Insects pre-treated with prohibitin and α-amylase dsRNA exhibited the reduction in mortality by 56.00–61.95 and 50.00–57.61% due to Cry1AcF toxin (in sub-lethal and lethal doses), respectively, compared to control. On the contrary, insects pre-treated with GLTP, ADAM and UDP-GT dsRNA did not show any significant decline in mortality due to Cry1AcF toxin, compared to control. A similar trend was observed for the non-receptor gene arylphorin. Our results propose the candidature of prohibitin and α-amylase as the functional receptors for Cry1AcF in *G. mellonella*. Other receptor-related genes such as GLTP, ADAM and UDP-GT may play redundant role in the Cry1AcF intoxication process assuming that the known receptors including CAD, ABCC2, ALP and APN1 can independently aid in Cry-induced toxicity. Additionally, the possibility of RNAi feedback regulation due to induced suppression of GLTP, ADAM and UDP-GT cannot be eliminated in our study because these genes may belong to multigene families. For example, UDP-GT may perform diverse physiological functions in different insects because order-specific gene diversification and inter-species sequence conservation for this multigene family was unraveled [29].

## Methods

### Insect rearing

*G. mellonella* larva (hatched from the eggs of our previous laboratory culture) were reared on an artificial diet containing wheat and corn flour, yeast extracts, milk powder, honey and glycerol at 26–28 °C and 60–65% relative humidity. Diet was supplemented with an antibiotic ampicillin at 20 mg per kg larval weight to inhibit the bacterial contamination. Fourth-instar larval stages (average body mass ~ 0.4 g) were surface sterilized with 70% ethanol for downstream experimental uses.

### RNA extraction from *G. mellonella*

To investigate the stage- and tissue-specific expression profile of various Cry receptor-like genes, the whole bodies of distinct life stages (first- to fifth-instar larvae) and diverse body parts (head, fat body, foregut, midgut and hindgut), were sampled by storing at—80 °C for RNA extraction. Following the manufacturer's recommendations, total RNA was extracted from various samples using TRIzol reagent (Invitrogen). To reduce genomic DNA contamination, isolated RNAs were digested with DNase I (TakaRa). Thermo Fisher Scientific's Nanodrop ND-1000 spectrophotometer was used to evaluate RNA purity, and RNA integrity was determined by resolving on a 1% (w/v) agarose gel. By using a first strand cDNA synthesis kit (Superscript VILO, Invitrogen), RNA (approximately 1 µg) from different life stages and body tissues was reverse transcribed to cDNA and kept at—20 °C for gene cloning purposes and RT-qPCR analyses.

### Cloning and in silico analysis of Cry receptor-like genes from *G. mellonella*

Local BLASTn (*E*-value cut-off < 1.0E^−30^) was employed to mine the homologous transcripts of different Cry receptor-related genes from *G. mellonella* non-redundant database in NCBI using known receptor genes of other insects as the query. *G. mellonella* homologs corresponding to various candidate genes were targeted by designing specific primers. Using a RACE cDNA amplification kit (Clontech, TaKaRa), 3' and 5'-RACE-Ready cDNAs were synthesized from the first strand cDNA (of insect midgut tissue) and primed by oligo(dT) primer and Smart II A oligonucleotides in accordance with the manufacturer’s protocol. 3'- and 5'-RACE fragments were amplified from the cDNA using sense and antisense gene-specific primers (GSPs), respectively, in addition to the usage of universal primers. PCR products were cloned in to pGEM-T Easy vector (Promega) and Sanger sequenced. A pair of primers was designed (at Primer3Plus online server; http://www.bioinformatics.nl/cgi-bin/primer3plus/) to verify the full-length cDNA sequence of individual receptor genes. Obtained sequences were deposited to the NCBI GenBank repository. Primer details are provided in Supplementary Table S3.

Different sequence features of the candidate genes were studied in FGENESH (https://www.softberry.com/) and Expasy (https://web.expasy.org/) webserver. Conserved domain and motif signatures were analyzed using NCBI conserved domain database (https://www.ncbi.nlm.nih.gov/Structure/cdd/) and InterProScan database (https://www.ebi.ac.uk/interpro/). SignalP 5.0 (http://www.cbs.dtu.dk/) and TMHMM 2.0 (https://services.healthtech.dtu.dk/) helped in signal peptide and transmembrane domain prediction, respectively. N- and O-glycosylated amino acid residues were searched in NetNGlyc 1.0 (http://www.cbs.dtu.dk/services/NetNGlyc/) and NetOGlyc 4.0 (http://www.cbs.dtu.dk/services/NetOGlyc/) webservers. Additionally, motif prediction of candidate receptors in *G. mellonella* and other insects was performed in MEME Suite 5.4.1 (https://meme-suite.org/meme/). Motifs were functionally annotated in HHpred (http://toolkit.tuebingen.mpg.de/hhpred) using default parameters.

Sequences were aligned with their homologs from different insect species via Clustal Omega MSA tool (https://www.ebi.ac.uk/Tools/msa/clustalo/). Construction of phylogenetic tree was performed in MEGA X tool; maximum likelihood method (using Le and Gascuel model and MODELTEST selection) was adopted to infer the evolutionary history. Each node in the tree consisted of more than 70% bootstrap value obtained from 1000 replicates.

The three-dimensional structures of Cry1AcF and different receptor proteins were modelled via homology modeling using SWISS-MODEL server. The obtained models were manually curated for precise sequence alignment with the query by employing the graphics program in Discovery Studio v. 14 (Biovia) [30–32]. Ligand-receptor docking analysis was carried out in “Macromolecules” module of Discovery Studio v. 14. ZDock algorithm was used which is a rigid-body protein–protein docking algorithm based on the Fast Fourier Transform correlation technique that is used to explore the rotational and translational space of a protein–protein system. The “Angular Step Size” value was set to 15 and the RMSD cut-off value was set as 4. After docking, the generated poses were scored and ranked according to their ZDock scores [33].

### RT-qPCR assay

RT-qPCR-based expression profile of different candidate genes was carried out in a CFX96 thermal cycler (BioRad). qPCR reaction mixture (10 μL) contained 1.5 ng cDNA, 750 nM each of sense and antisense primers, and 5 μL of SYBR Green PCR master-mix (BioRad). qPCR amplification condition were followed as: a hot start of 95 °C for 30 s, followed by 40 cycles of 95 °C for 10 s and 60 °C for 30 s. In addition, a melt curve program (95 °C for 15 s, 60 °C for 15 s, followed by a slow ramp from 60 to 95 °C) was followed to determine the specificity of amplification. Quantification cycle (Cq) values were obatained from CFX Maestro software (BioRad). Two housekeeping genes of *G. mellonella*, i.e. *18S rRNA* and *EF-1α*, were included as the internal reference for normalization of gene expression data. Fold change in expression was determined using 2^−ΔΔCq^ method. qPCR was performed with five biological and three technical replicates for each samples. Primers designed in OligoAnalyzer tool (https://eu.idtdna.com/). PCR reaction efficiency of various primers was calculated via generating a standard curve (Cq values versus cDNA concentrations) from five-fold dilution series of cDNA samples followed by determination of slope using a linear regression equation: *E* = (10^(−1/slope)^ – 1) × 100. Primer details and PCR efficiency are provided in Supplementary Table S4.

### Synthesis of target gene dsRNA

Initially, target dsRNA molecules were designed using various in silico tools including siDirect (http://sidirect2.rnai.jp/), Dharmacon (http://horizondiscovery.com/) and dsCheck (http://dscheck.rnai.jp/) as these tools predict the siRNA formation probability and aid in averting off-target effects by analyzing sequence homology of predicted siRNAs with that of non-target species. DsRNA-binding regions of receptor genes were PCR-amplified (by following standard protocol) from the cDNA (isolated from fourth-instar *G. mellonella* midgut tissue) using gene-specific primers that contained *Sac*I and *Hind*III restriction endonuclease sites (Supplementary Table S4). PCR products were ligated onto *Sac*I and *Hind*III-digested L4440 plasmid (Addgene), which contains two T7 polymerase promoters (drive transcription of DNA to RNA) in inverted orientation flanking the multiple cloning site. For bacterial expression of dsRNA, *E. coli* HT115(DE3) cells were transformed with recombinant L4440. HT115 cells were grown in LB medium supplemented with antibiotics ampicillin (50 µg mL^−1^) and tetracycline (12.5 µg mL^−1^) at 37 °C for 12 h with constant stirring at 200 rpm. Next, 0.4 mM IPTG was added to the culture (to induce T7 polymerase synthesis) followed by incubation for additional 4 h at 37 °C. Expressed dsRNAs were extracted from bacterial aliquots (cells precipitated by centrifugation at 5000 × g for 10 min at 4 °C and re-suspended in 0.05 M PBS at 10:1 ratio) and checked via electrophoresis on a 1% (w/v) agarose gel. A *gfp* gene (Genbank ID: HF675000) cloned in L4440 was used as the control.

### RNAi bioassay

20 µL solution of HT115 cells expressing target dsRNA or GFP dsRNA (equivalent to approximately 10 µg of dsRNA) or 0.05 M PBS (negative control) was force-fed to a 12 h starved fourth-instar *G. mellonella* larvae via a sterilized 26-gauge hypodermic needle. Next, insects were randomly placed into sterilized 6-well polystyrene tissue culture plates containing artificial diets and plates were incubated at 28 °C in dark. After 24 h of dsRNA delivery, insects were again force-fed with 20 µL of PBS containing Cry1AcF toxin (in LD_50_ and LD_90_ doses) using a sterilized needle; PBS was used as the negative control. Insects were incubated in 6-well plates containing artificial diet at 28 °C and after 24 h of Cry ingestion, larval mortality data was recorded. The whole experiment was repeated five times (*n* = 50). The details of Cry1AcF protein production and purification are depicted in Rathinam et al. [23] and patent number 237912.

Induced silencing of target receptor genes was ascertained by RT-qPCR analyses. RNA was extracted from the midgut samples of ten random insects corresponding to each dsRNA treatment groups and reverse-transcribed to cDNA as explained above. qPCR amplification conditions were followed as described above.

### Statistical analysis

Bioassay and RT-qPCR data were subjected to one-way or two-way analysis of variance (ANOVA) test followed by Tukey’s honest significant difference (HSD) test in SAS software (version 14.1). Statistical comparisons were performed either between different treatments or individually compared to control as described in the respective figure legends.

## Supplementary Information

**Additionalfile 1: Supplementary Figure S1.** Protein-protein interaction between Cry1AcF ligand and known gut receptors CAD, ABCC2, ALP and APN1. Cry1AcF bound with these receptors via a number of Pi interactions, hydrogen bonds and salt bridges. The ZDock scores (greater value indicates greater contact surface area between ligand and receptor) for Cry-CAD, Cry-ABCC2, Cry-ALP and Cry-APN1 complexes were 2321, 1907, 2189 and 2060 Å^2^, respectively. Domain I, II and III of Cry1AcF arehighlighted in magenta, ochre yellow and green color, respectively. **Supplementary Figure S2.** Evolutionary relationship of ADAM protein from *G. mellonella* with their corresponding homologues from other insect species. The phylogenetic treewas constructed in MEGA X software using Maximum Likelihood method; best model was selected via MODELTEST using Le and Gascuel method. Bootstrap consensus was inferred from 1000 replicates and branches corresponding to < 70% replicates were collapsed. The analyses included 61 amino acid sequences. NCBI accessionnumbers of different entries are provided in parentheses. All gaps and missingdata positions were eliminated after sequence alignment. *Homo sapiens* sequencefor the corresponding protein was used as the out-group (marked with ●), and *G. mellonella* entry is indicated in bold font. Entries in blue, black, red and green correspond to the members of the order Hymenoptera, Isoptera, Coleoptera and Lepidoptera, respectively. **Supplementary Figure S3.** Evolutionary relationship of prohibitin protein from *G. mellonella* with their corresponding homologues from other insect species. The phylogenetic tree was constructed in MEGA X software using Maximum Likelihood method; best model was selected via MODELTEST using Le and Gascuel method. Bootstrap consensus was inferred from 1000 replicates and branches corresponding to < 70% replicates were collapsed. The analyses included 88 amino acid sequences. NCBI accession numbers ofdifferent entries are provided in parentheses. All gaps and missing data positions were eliminated after sequence alignment. *Homo sapiens* sequence for the corresponding protein was used as the out-group (marked with ●), and *G. mellonella* entry is indicated in bold font. Entries in red, teal, green, black,blue, purple, fuchsia, olive, maroon, grey and aqua correspond to the membersof the order Lepidoptera, Phasmatodea, Dictyoptera, Psocoptera, Coleoptera, Hemiptera, Zygentoma, Collembola, Hymenoptera, Siphonaptera and Diptera,respectively. **Supplementary Figure S4.** Evolutionary relationship of GLTP protein from *G. mellonella* with their corresponding homologues from insect species of different orders. The phylogenetic tree was constructed in MEGA X software using Maximum Likelihood method; best model was selected via MODELTEST using Le and Gascuel method. Bootstrap consensus was inferred from 1000 replicates and branches corresponding to < 50% replicates were collapsed. The analyses included 77 amino acid sequences. A discrete Gamma distribution was used to model evolutionary rate differences among sites [5 categories (+G, parameter = 2.2830)]. Initial tree(s) for the heuristic search were obtained by applying the Neighbour-Joining method to a matrix of pairwise distances estimated using a JTT model, and then selecting the topology with superior log likelihood value). NCBI accession numbers of different entries are provided in parentheses. All gaps and missing data positions were eliminated after sequence alignment. *Homo sapiens *sequence for the corresponding protein was used as theout-group (marked with ●), and *G. mellonella* entry is indicated in bold font. Entries in blue, black, green, purple, teal, fuchsia and red correspond to the representative members of orders Hymenoptera, Thysanoptera, Ephemeroptera, Isoptera, Hemiptera, Phasmatodea and Lepidoptera, respectively. **Supplementary Figure S5. **Evolutionary relationship of arylphorin protein from *G. mellonella* with their corresponding homologues from insect species of different orders. The phylogenetic tree was constructed in MEGA X software using Maximum Likelihood method; best model was selected via MODELTEST using Le and Gascuel method. Bootstrap consensus was inferred from 1000 replicates and branches corresponding to < 50% replicates were collapsed. The analyses included 45 amino acid sequences. A discrete Gamma distribution was used to model evolutionary rate differences among sites [5 categories (+G, parameter =1.4705)]. Initial tree(s) for the heuristic search were obtained by applying the Neighbour-Joining method to a matrix of pairwise distances estimated using a JTT model, and then selecting the topology with superior log likelihood value). NCBI accession numbers of different entries are provided in parentheses. All gaps and missing data positions were eliminated after sequence alignment. *G. mellonella* entry is indicated in bold font. Entries in blue, black, red and green correspond to the representative members of orders Lepidoptera, Siphonaptera and Diptera, respectively. No outgroup has been used inthe analyses because arylphorin orthologue was not found in *Homo sapiens*. **Supplementary Figure S6.** Evolutionary relationship of UDP-GT protein from *G. mellonella* with their corresponding homologues from insect species of order Lepidoptera. The phylogenetic tree was constructed in MEGA X software using Maximum Likelihood method; best model was selected via MODELTEST using Le and Gascuel method. Bootstrap consensus was inferred from 1000 replicates and branches corresponding to < 50% replicates were collapsed. The analyses included 24 amino acid sequences. A discrete Gamma distribution was used to model evolutionary rate differences among sites [5 categories (+G, parameter =1.3839)]. Initial tree(s) for the heuristic search were obtained by applying the Neighbour-Joining method to a matrix of pairwise distances estimated using a JTT model, and then selecting the topology with superior log likelihood value). NCBI accession numbers of different entries are provided in parentheses. All gaps and missing data positions were eliminated after sequence alignment. *Homo sapiens* sequence for the corresponding protein was used as the out-group (marked with ●), and *G. mellonella* entry is indicated in bold font. **Supplementary Figure S7.** Evolutionary relationship of α-amylase protein from *G. mellonella* with their corresponding homologues from insect species of order Lepidoptera. The phylogenetic tree was constructed in MEGA X software using Maximum Likelihood method; best model was selected via MODELTEST using Le and Gascuel method. Bootstrap consensus was inferred from 1000 replicates and branches corresponding to < 70% replicates were collapsed. The analyses included 37 amino acid sequences. A discrete Gamma distribution was used to model evolutionary rate differences among sites [5 categories (+G, parameter =0.9598)]. Initial tree(s) for the heuristic search were obtained by applying the Neighbour-Joining method to a matrix of pairwise distances estimated using a JTT model, and then selecting the topology with superior log likelihood value). NCBI accession numbers of different entries are provided in parentheses. All gaps and missing data positions were eliminated after sequence alignment. *Homo sapiens* sequence for the corresponding protein was used as the out-group (marked with ●), and *G. mellonella* entry is indicated in bold font. **Supplementary Figure S8.** The conserved motif distribution of ADAM proteins across the different insect orders. Each categorized motif logo generated by MEME is displayed in differentially colored boxes. Legend (at the bottom) depicts the protein sequence of corresponding motifs. Motifs were serially numbered according to their frequency of occurrence in MEME bioinformatics tool. **Supplementary Figure S9.** The conserved motif distribution of prohibitin proteins across the different insect orders. Each categorized motif logo generated by MEME is displayed in differentially colored boxes. Legend (at the bottom right) depicts the protein sequence of corresponding motifs. Motifs were serially numbered according to their frequency of occurrence in MEME bioinformatics tool. **Supplementary Figure S10.** The conserved motif distribution of UDP-GT proteins across the different insect orders. Each categorized motif logo generated by MEME is displayed in differentially colored boxes. Legend (at the bottom) depicts the protein sequence of corresponding motifs. Motifs were serially numbered according to their frequency of occurrence in MEME bioinformatics tool. **Supplementary Figure S11.** The conserved motif distribution of α-amylase proteins across the different insect orders. Each categorized motif logo generated by MEME is displayed in differentially colored boxes. Legend (at the bottom right) depicts the protein sequence of corresponding motifs. Motifs were serially numbered according to their frequency of occurrence in MEME bioinformatics tool. **Supplementary Figure S12.** The conserved motif distribution of arylphorin proteins across the different insect orders. Each categorized motif logo generated by MEME is displayed in differentially colored boxes. Legend (at the right hand side) depicts the protein sequence of corresponding motifs. Motifs were serially numbered according to their frequency of occurrence in MEME bioinformatics tool. **Supplementary Figure S13.** The conserved motif distribution of GLTP proteins across the different insect orders. Each categorized motif logo generated by MEME is displayed in differentially colored boxes. Legend (at the bottom right) depicts the protein sequence of corresponding motifs. Motifs were serially numbered according to their frequency of occurrence in MEME bioinformatics tool.

**Additional file 1: Supplementary Table S1.** A summary of number of unigenes corresponding to different Cry toxin receptors from the NCBI non redundant database for *G. mellonella* transcripts/ESTs (E-value cut off < 1.0E-30). **Supplementary Table S2.** In silico Protein-Protein Interface analysis. **Supplementary Table S3.** Oligonucleotides used for RACE-PCR and verifying full-length cDNAsequence. Tm = 60°C. **Supplementary Table S4.** Oligonucleotides used for RNAi and RT-qPCR analysis. Tm = 60°C.

## Data Availability

The data sets supporting this article are included in the article and in the additional files. The NCBI Genbank accession numbers for the cloned genes are MZ561435, MZ561436, MZ576274, MZ576275, MZ576276 and MZ576277.

## Abbreviations

Bt: *Bacillus thuringiensis*
Cry: Crystal
CAD: Cadherin
ABCC: ABC transporter subfamily C
ALP: Alkaline phosphatase
APN: Alanyl aminopeptidase N
GLTP: Glycolipid transfer protein
UDP-GT: Uridine diphosphate-glucosyltransferase
ADAM: ADAM metalloprotease
RNAi: RNA interference
RT-qPCR: Reverse transcription – quantitative polymerase chain reaction
LD_50_: Lethal dose 50
C-terminal: Carboxy terminus
N-terminal: Amino terminus
ORF: Open reading frame
NCBI: National center for biotechnology information
pI: Isoelectric point
aa: Amino acids
bp: Base pair
GPI: Glycosylphosphatidylinositol
GalNAc: N-acetylgalactoseamine
mRNA: Messenger RNA
RACE: Rapid amplification of cDNA ends
