# Supplementary material for: RNAi-mediated knockdown of gut receptor-like genes prohibitin and α-amylase altered the susceptibility of Galleria mellonella to Cry1AcF toxin
Source: BMC Genomics. 2022 Aug 18;23:601. doi: 10.1186/s12864-022-08843-8 (PMC9389788; doi:10.1186/s12864-022-08843-8)

SUPPLEMENTARY FIGURES

Title: **RNAi-mediated knockdown of gut receptors prohibitin and UDP-GT altered the susceptibility of *Galleria mellonella* to Cry1AcF toxin**

Authors: Tushar K. Dutta[1*], Abhishek Mandal[2], Artha Kundu[1], Victor Phani[3], Chetna Mathur[1], Arudhimath Veeresh[1], Rohini Sreevathsa[4]

Affiliation: [1]Division of Nematology, ICAR-Indian Agricultural Research Institute, New Delhi, 110012, India
[2]Division of Agricultural Chemicals, ICAR-Indian Agricultural Research Institute, New Delhi, 110012, India
[3]Department of Agricultural Entomology, College of Agriculture, Uttar Banga Krishi Viswavidyalaya, Dakshin Dinajpur, West Bengal, India
[4]ICAR-National Institute for Plant Biotechnology, New Delhi, 110012, India

Corresponding Author
[*]Dr. Tushar K Dutta
Division of Nematology
ICAR-Indian Agricultural Research Institute
New Delhi, India-110012
TEL: +91-11-2584-2721
Email: tushar.dutta@icar.gov.in; nemaiari@gmail.com

Supplementary Figure S1. Protein-protein interaction between Cry1AcF ligand and known gut receptors CAD, ABCC2, ALP and APN1. Cry1AcF bound with these receptors via a number of Pi interactions, hydrogen bonds and salt bridges. The ZDock scores (greater value indicates greater contact surface area between ligand and receptor) for Cry-CAD, Cry-ABCC2, Cry-ALP and Cry-APN1 complexes were 2321, 1907, 2189 and 2060 Å^2, respectively. Domain I, II and III of Cry1AcF are highlighted in magenta, ochre yellow and green color, respectively.

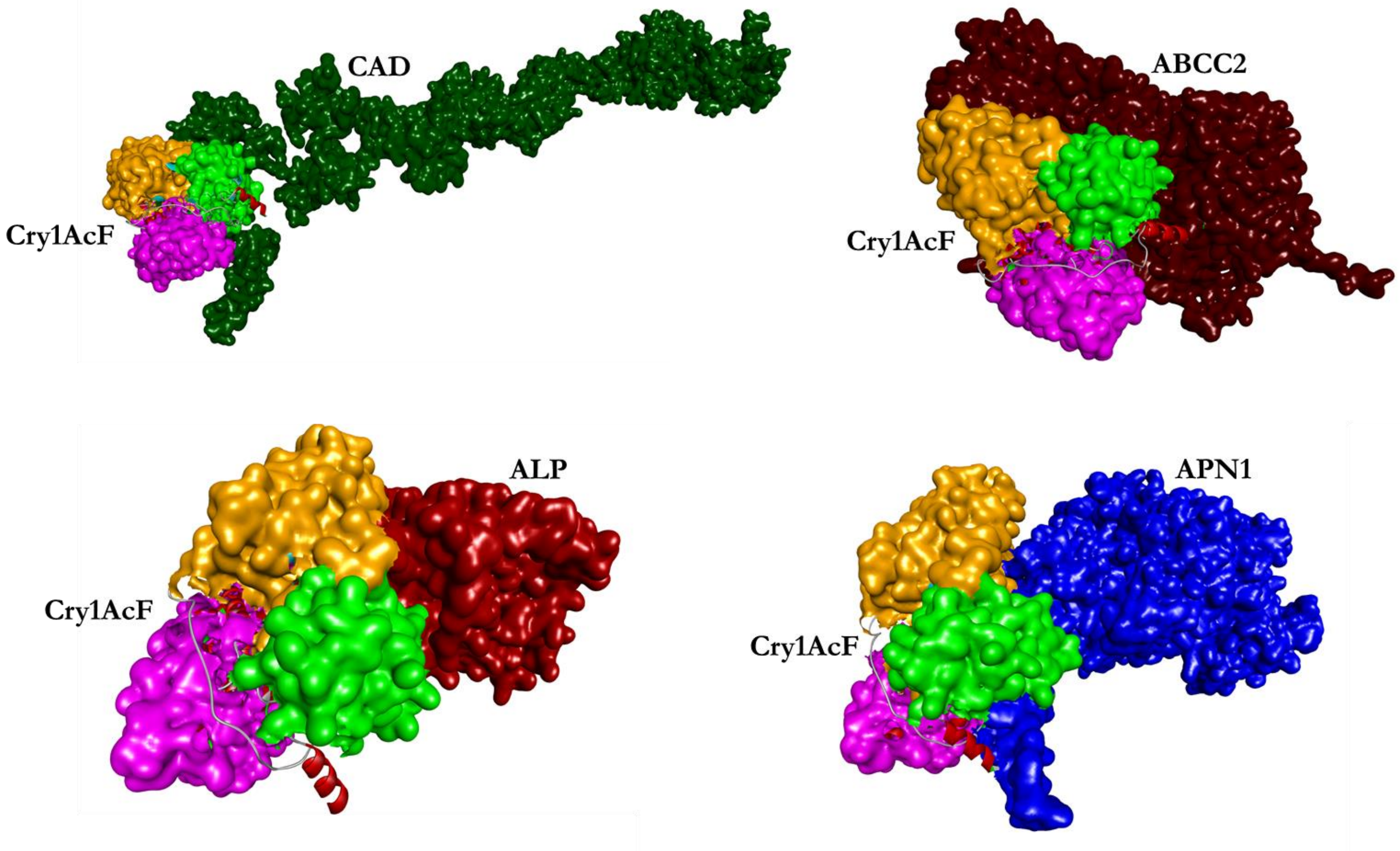

Supplementary Figure S2. Evolutionary relationship of ADAM protein from *G. mellonella* with their corresponding homologues from other insect species. The phylogenetic tree was constructed in MEGA X software using Maximum Likelihood method; best model was selected via MODELTEST using Le and Gascuel method. Bootstrap consensus was inferred from 1000 replicates and branches corresponding to < 70% replicates were collapsed. The analyses included 61 amino acid sequences. NCBI accession numbers of different entries are provided in parentheses. All gaps and missing data positions were eliminated after sequence alignment. *Homo sapiens* sequence for the corresponding protein was used as the out-group (marked with ●), and *G. mellonella* entry is indicated in bold font. Entries in blue, black, red and green correspond to the members of the order Hymenoptera, Isoptera, Coleoptera and Lepidoptera, respectively.

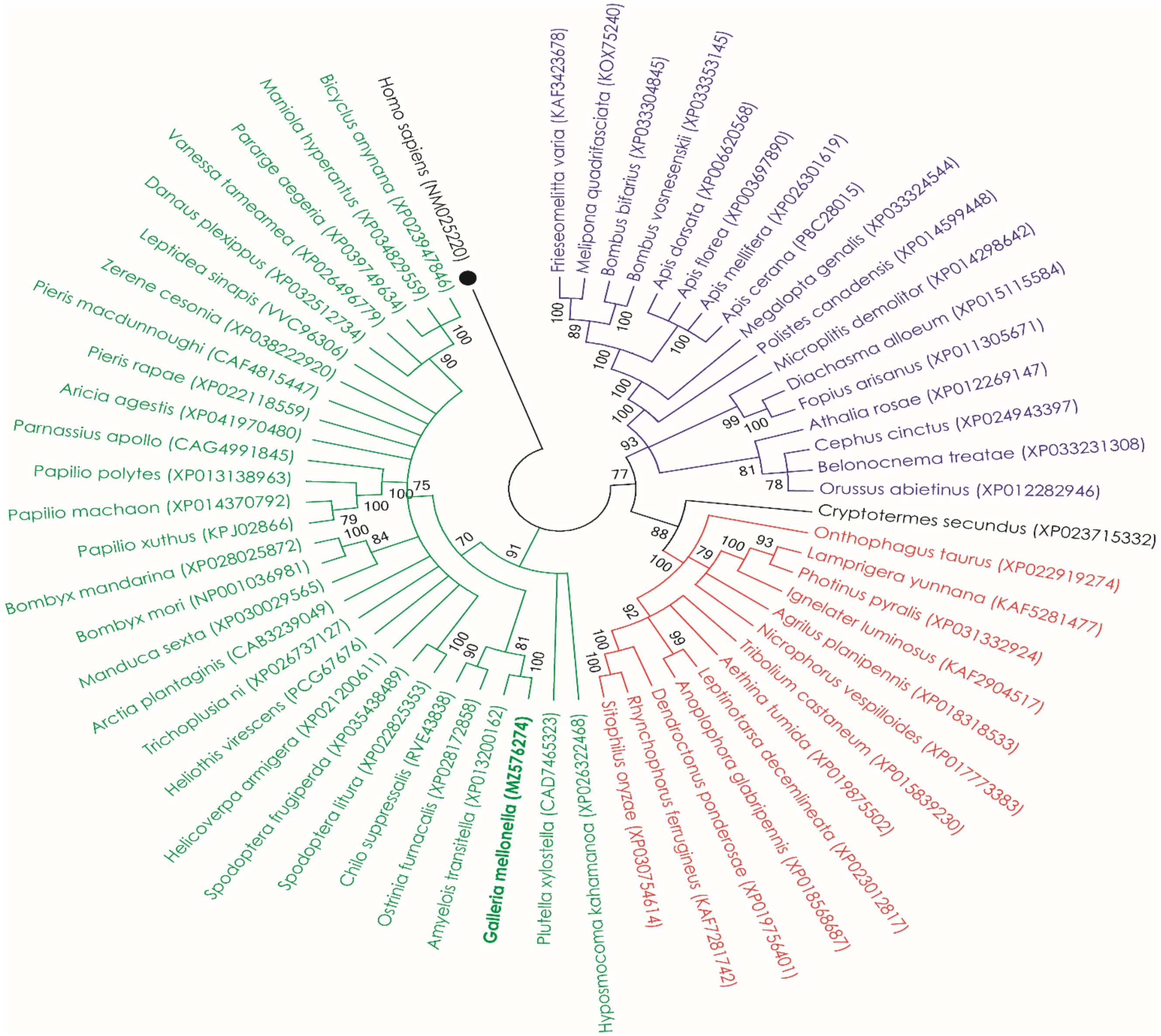

Supplementary Figure S3. Evolutionary relationship of prohibitin protein from *G. mellonella* with their corresponding homologues from other insect species. The phylogenetic tree was constructed in MEGA X software using Maximum Likelihood method; best model was selected via MODELTEST using Le and Gascuel method. Bootstrap consensus was inferred from 1000 replicates and branches corresponding to < 70% replicates were collapsed. The analyses included 88 amino acid sequences. NCBI accession numbers of different entries are provided in parentheses. All gaps and missing data positions were eliminated after sequence alignment. *Homo sapiens* sequence for the corresponding protein was used as the out-group (marked with ●), and *G. mellonella* entry is indicated in bold font. Entries in red, teal, green, black, blue, purple, fuchsia, olive, maroon, grey and aqua correspond to the members of the order Lepidoptera, Phasmatodea, Dictyoptera, Psocoptera, Coleoptera, Hemiptera, Zygentoma, Collembola, Hymenoptera, Siphonaptera and Diptera, respectively.

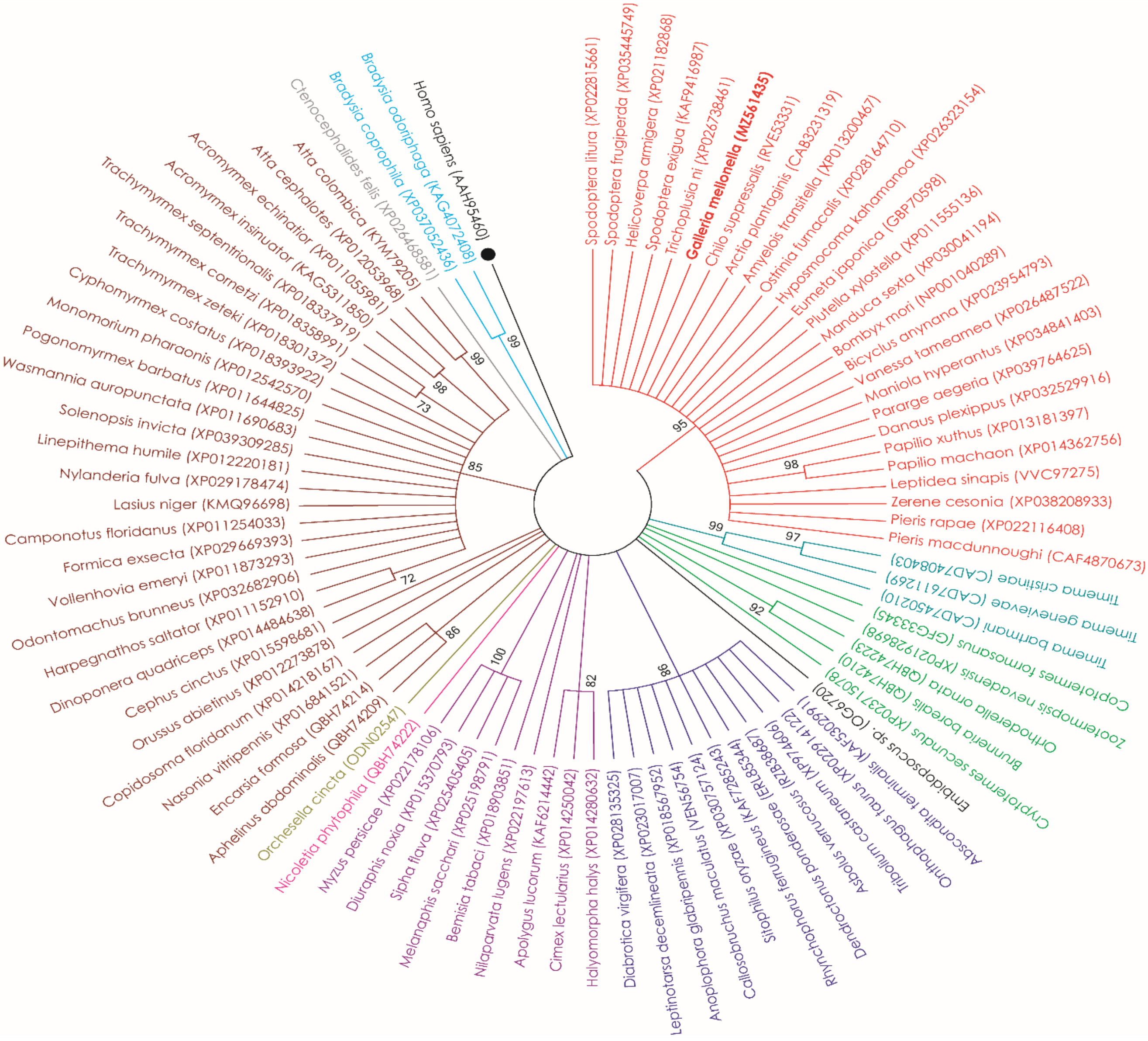

Supplementary Figure S4. Evolutionary relationship of GLTP protein from *G. mellonella* with their corresponding homologues from insect species of different orders. The phylogenetic tree was constructed in MEGA X software using Maximum Likelihood method; best model was selected via MODELTEST using Le and Gascuel method. Bootstrap consensus was inferred from 1000 replicates and branches corresponding to < 50% replicates were collapsed. The analyses included 77 amino acid sequences. A discrete Gamma distribution was used to model evolutionary rate differences among sites [5 categories (+*G*, parameter = 2.2830)]. Initial tree(s) for the heuristic search were obtained by applying the Neighbour-Joining method to a matrix of pairwise distances estimated using a JTT model, and then selecting the topology with superior log likelihood value). NCBI accession numbers of different entries are provided in parentheses. All gaps and missing data positions were eliminated after sequence alignment. *Homo sapiens* sequence for the corresponding protein was used as the out-group (marked with ●), and *G. mellonella* entry is indicated in bold font. Entries in blue, black, green, purple, teal, fuchsia and red correspond to the representative members of orders Hymenoptera, Thysanoptera, Ephemeroptera, Isoptera, Hemiptera, Phasmatodea and Lepidoptera, respectively.

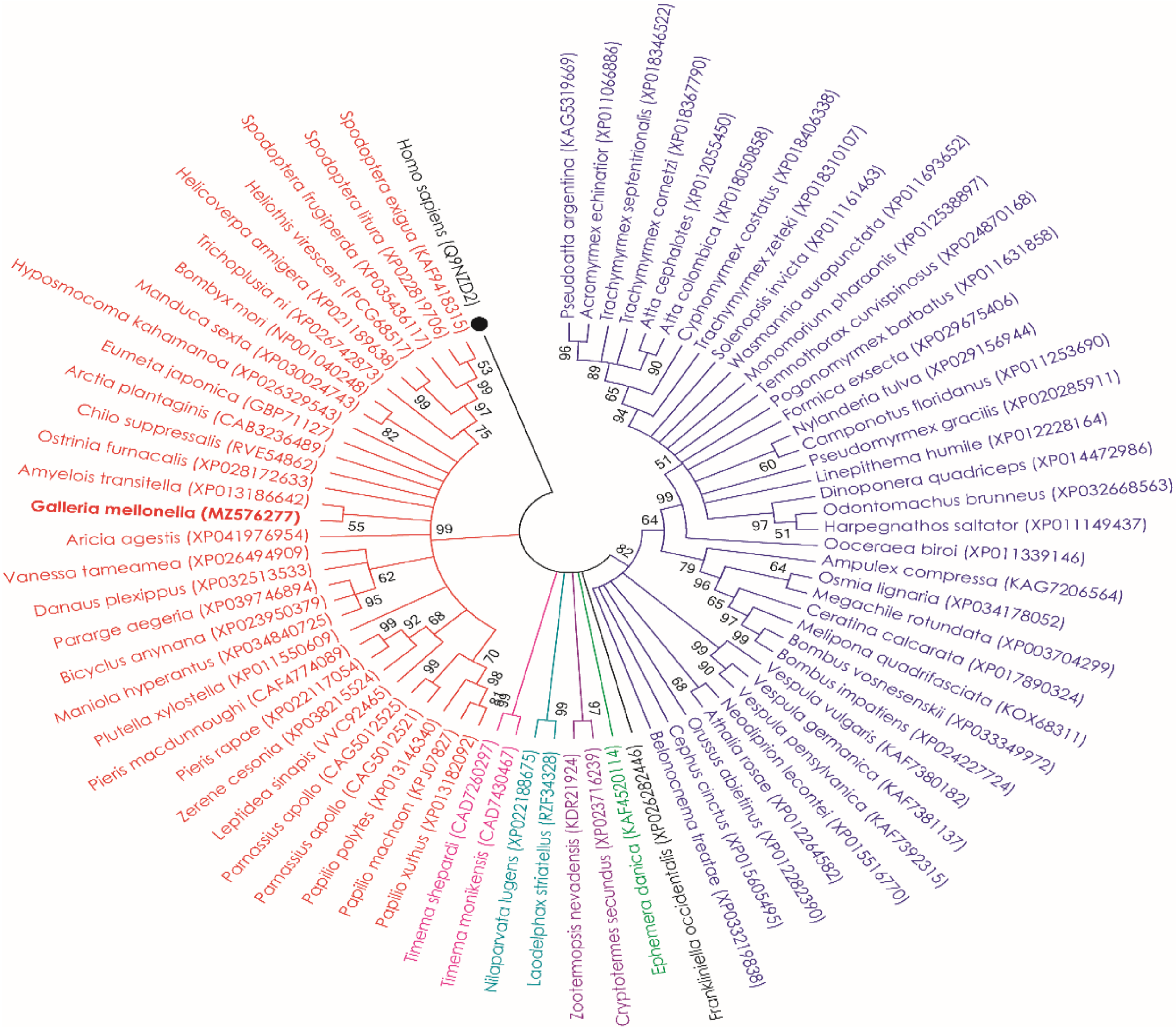

Supplementary Figure S5. Evolutionary relationship of arylphorin protein from *G. mellonella* with their corresponding homologues from insect species of different orders. The phylogenetic tree was constructed in MEGA X software using Maximum Likelihood method; best model was selected via MODELTEST using Le and Gascuel method. Bootstrap consensus was inferred from 1000 replicates and branches corresponding to < 50% replicates were collapsed. The analyses included 45 amino acid sequences. A discrete Gamma distribution was used to model evolutionary rate differences among sites [5 categories (+*G*, parameter = 1.4705)]. Initial tree(s) for the heuristic search were obtained by applying the Neighbour-Joining method to a matrix of pairwise distances estimated using a JTT model, and then selecting the topology with superior log likelihood value). NCBI accession numbers of different entries are provided in parentheses. All gaps and missing data positions were eliminated after sequence alignment. *G. mellonella* entry is indicated in bold font. Entries in blue, black, red and green correspond to the representative members of orders Lepidoptera, Siphonaptera and Diptera, respectively. No outgroup has been used in the analyses because arylphorin orthologue was not found in *Homo sapiens*.

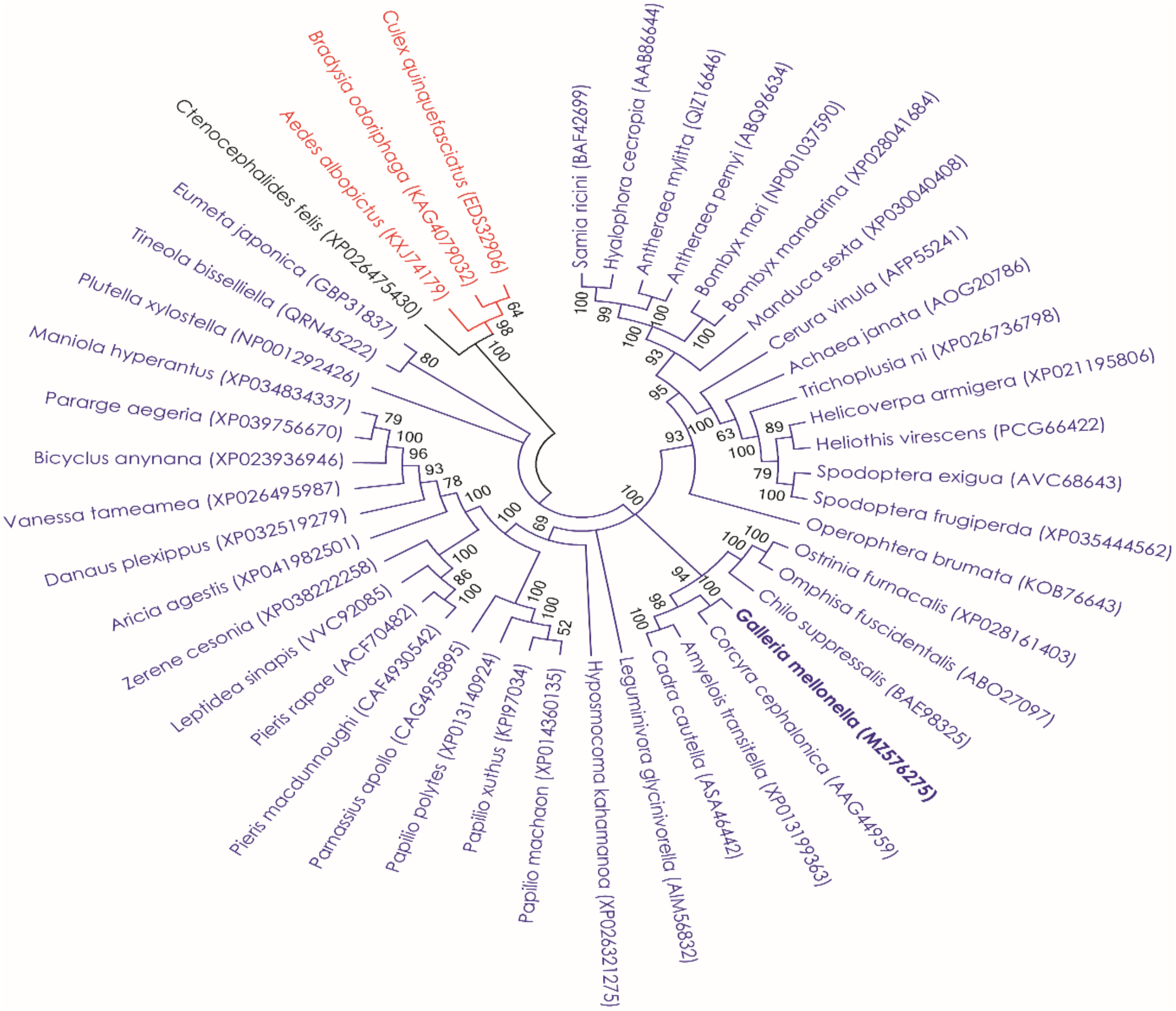

Supplementary Figure S6. Evolutionary relationship of UDP-GT protein from *G. mellonella* with their corresponding homologues from insect species of order Lepidoptera. The phylogenetic tree was constructed in MEGA X software using Maximum Likelihood method; best model was selected via MODELTEST using Le and Gascuel method. Bootstrap consensus was inferred from 1000 replicates and branches corresponding to < 50% replicates were collapsed. The analyses included 24 amino acid sequences. A discrete Gamma distribution was used to model evolutionary rate differences among sites [5 categories (+*G*, parameter = 1.3839)]. Initial tree(s) for the heuristic search were obtained by applying the Neighbour-Joining method to a matrix of pairwise distances estimated using a JTT model, and then selecting the topology with superior log likelihood value). NCBI accession numbers of different entries are provided in parentheses. All gaps and missing data positions were eliminated after sequence alignment. *Homo sapiens* sequence for the corresponding protein was used as the out-group (marked with ●), and *G. mellonella* entry is indicated in bold font.

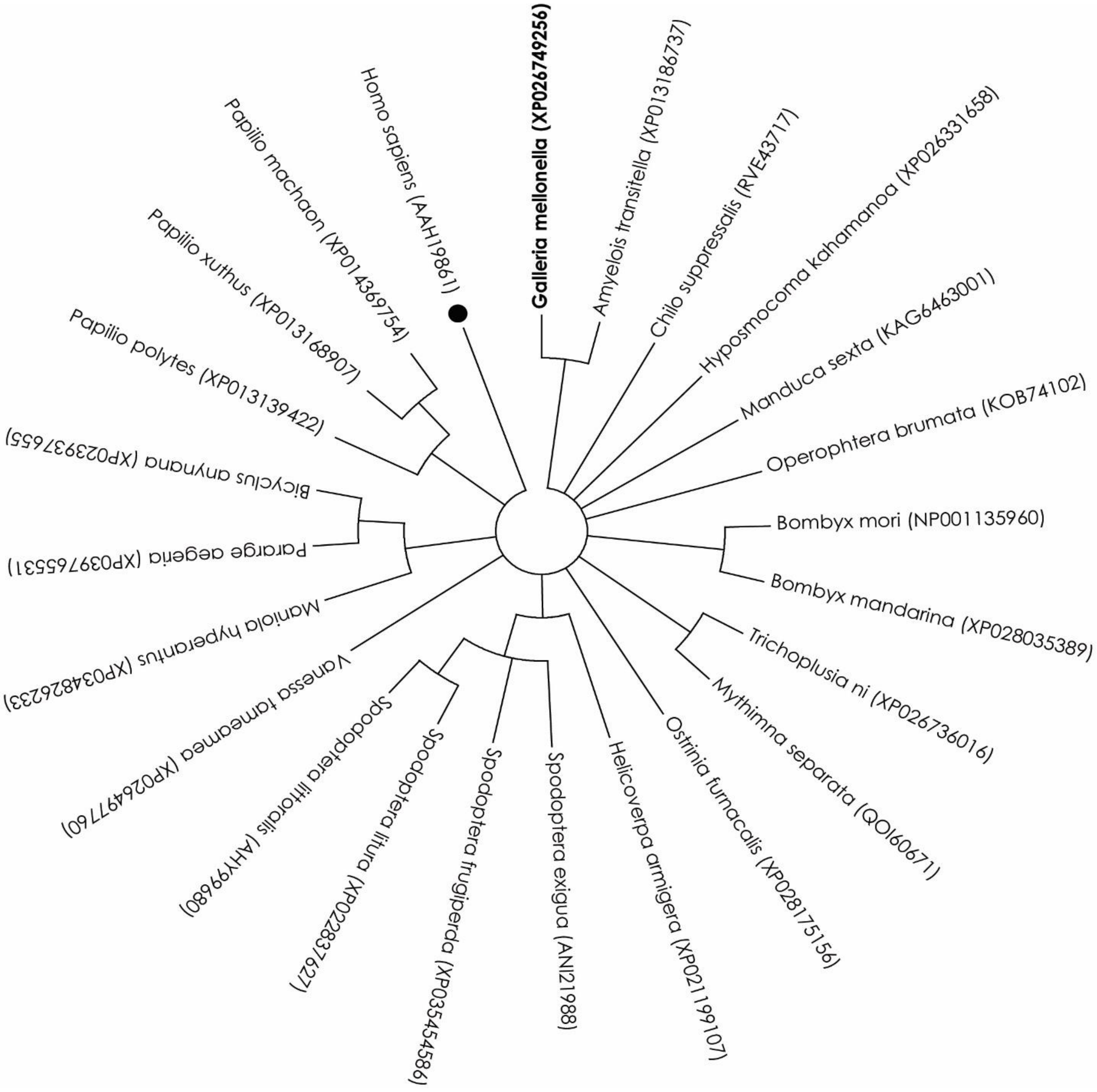

Supplementary Figure S7. Evolutionary relationship of α-amylase protein from *G. mellonella* with their corresponding homologues from insect species of order Lepidoptera. The phylogenetic tree was constructed in MEGA X software using Maximum Likelihood method; best model was selected via MODELTEST using Le and Gascuel method. Bootstrap consensus was inferred from 1000 replicates and branches corresponding to < 70% replicates were collapsed. The analyses included 37 amino acid sequences. A discrete Gamma distribution was used to model evolutionary rate differences among sites [5 categories (+*G*, parameter = 0.9598)]. Initial tree(s) for the heuristic search were obtained by applying the Neighbour-Joining method to a matrix of pairwise distances estimated using a JTT model, and then selecting the topology with superior log likelihood value). NCBI accession numbers of different entries are provided in parentheses. All gaps and missing data positions were eliminated after sequence alignment. *Homo sapiens* sequence for the corresponding protein was used as the out-group (marked with ●), and *G. mellonella* entry is indicated in bold font.

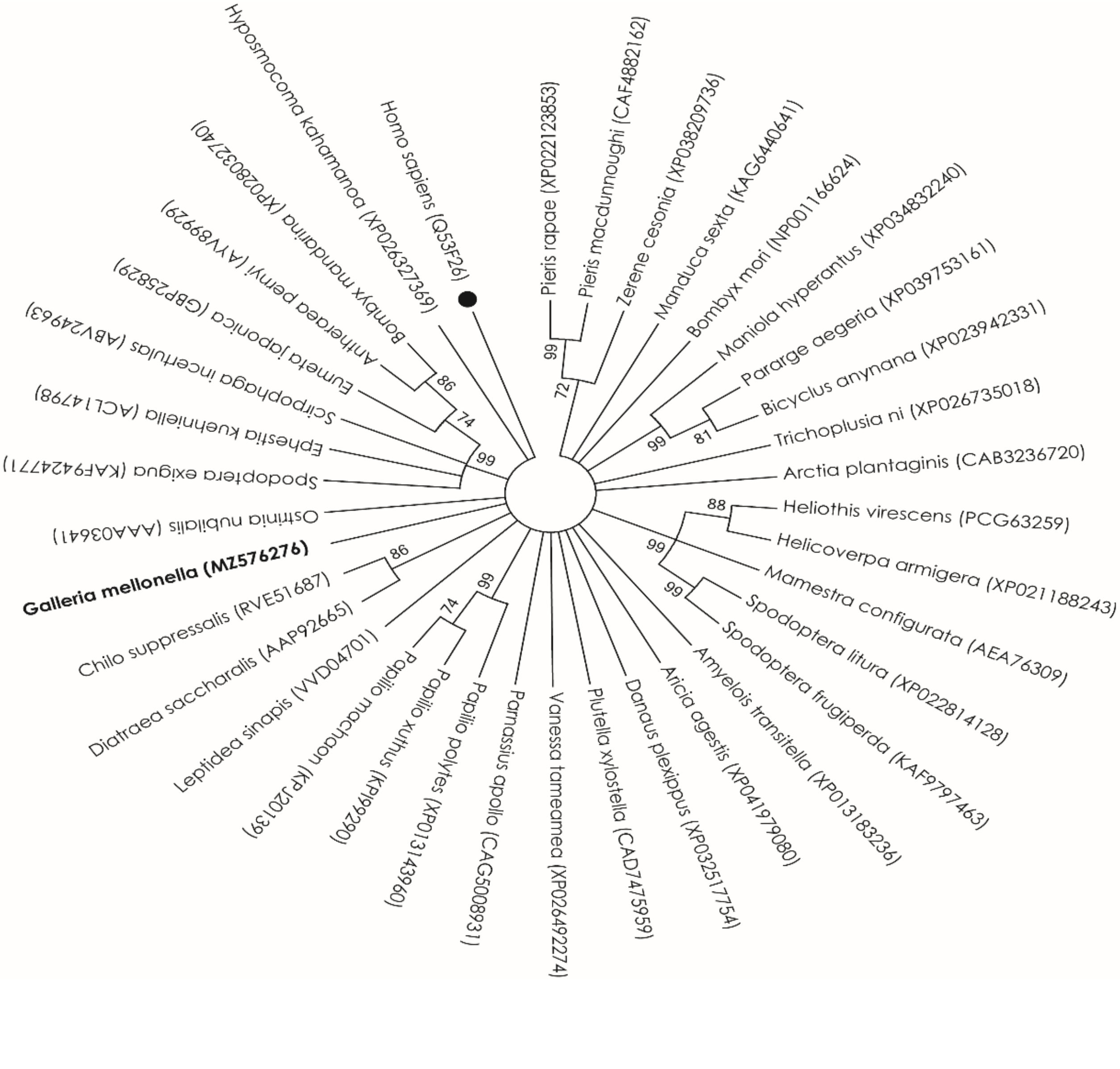

Supplementary Figure S8. The conserved motif distribution of ADAM proteins across the different insect orders. Each categorized motif logo generated by MEME is displayed in differentially colored boxes. Legend (at the bottom) depicts the protein sequence of corresponding motifs. Motifs were serially numbered according to their frequency of occurrence in MEME bioinformatics tool.

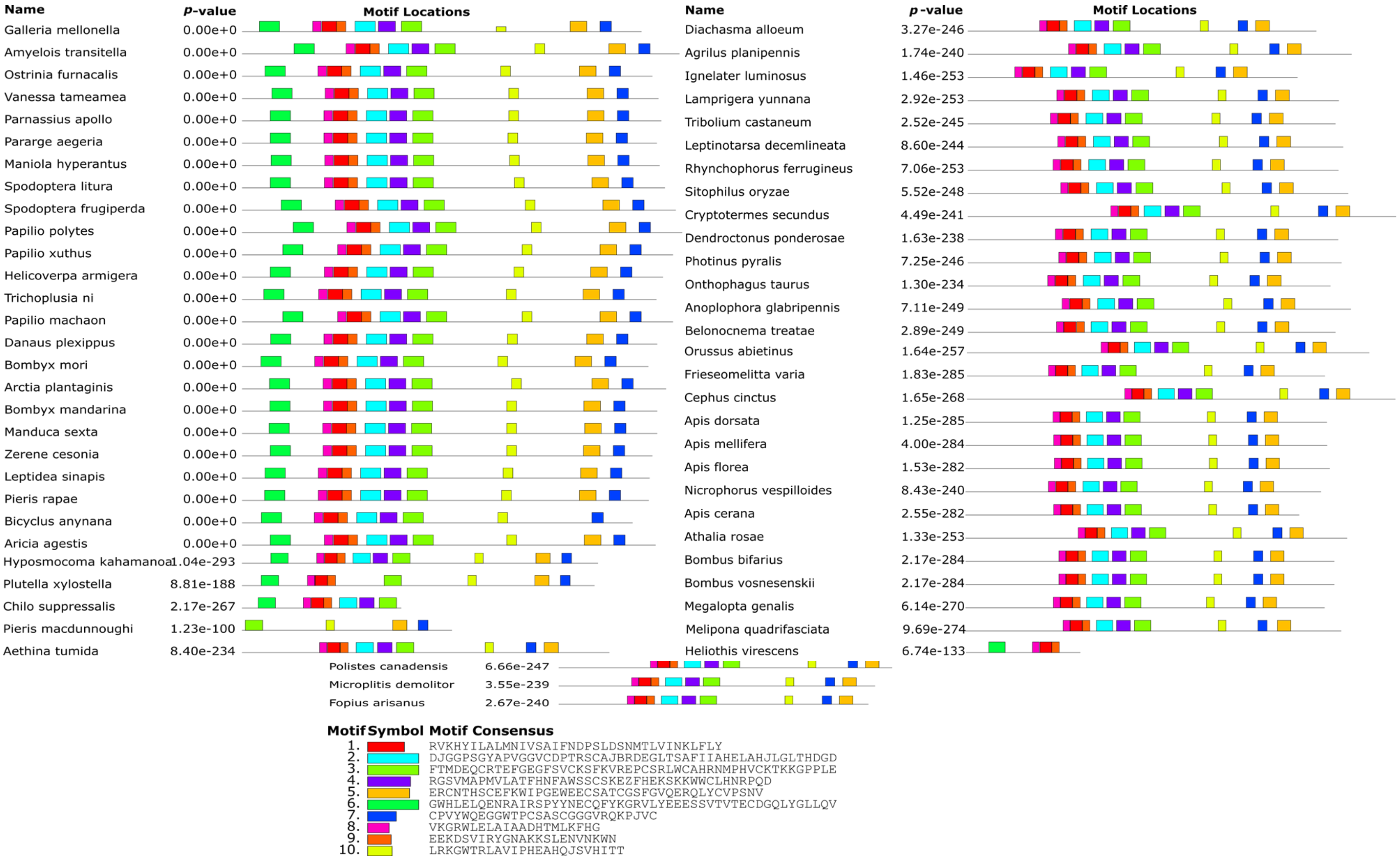

Supplementary Figure S9. The conserved motif distribution of prohibitin proteins across the different insect orders. Each categorized motif logo generated by MEME is displayed in differentially colored boxes. Legend (at the bottom right) depicts the protein sequence of corresponding motifs. Motifs were serially numbered according to their frequency of occurrence in MEME bioinformatics tool.

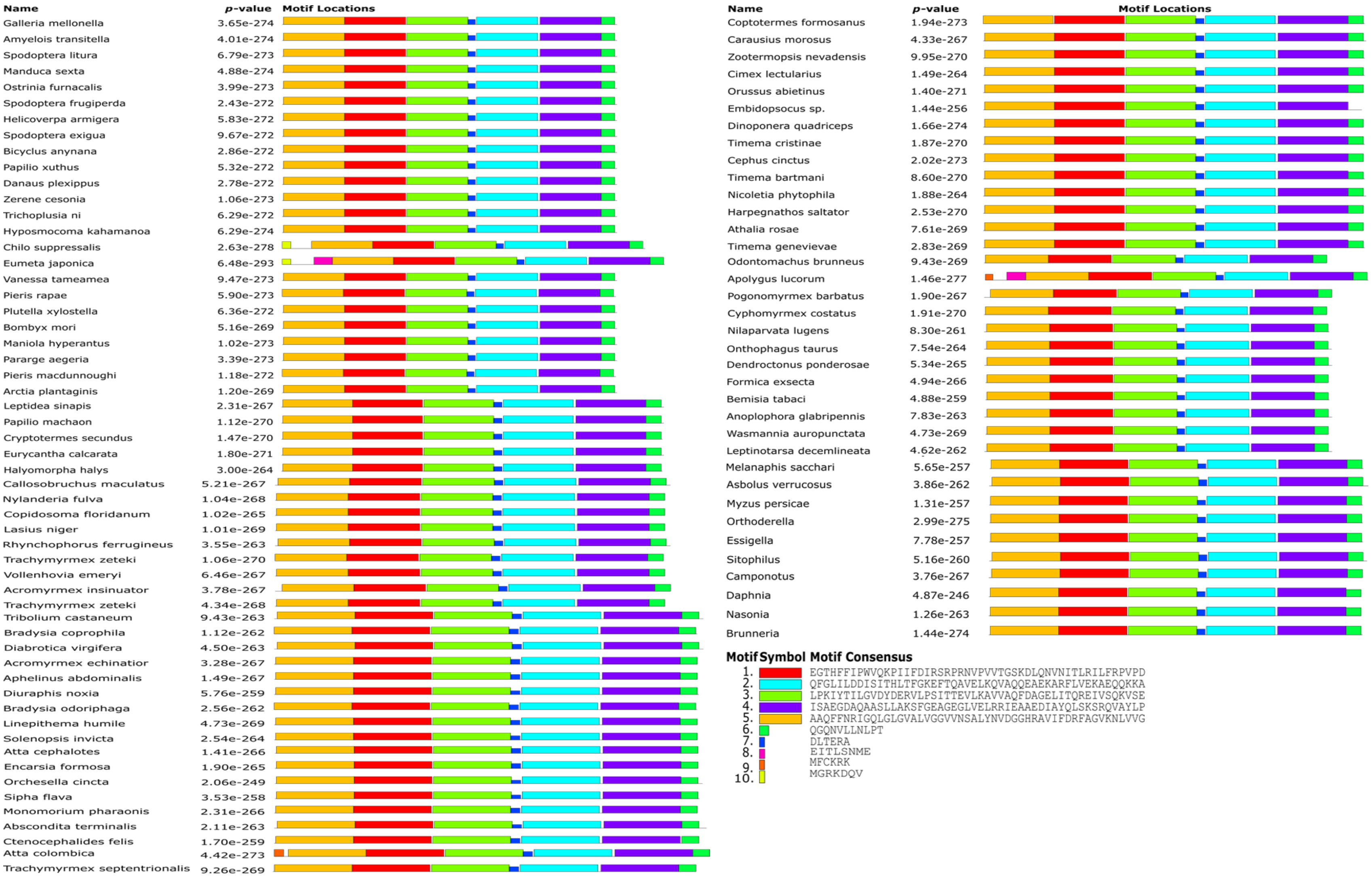

Supplementary Figure S10. The conserved motif distribution of UDP-GT proteins across the different insect orders. Each categorized motif logo generated by MEME is displayed in differentially colored boxes. Legend (at the bottom) depicts the protein sequence of corresponding motifs. Motifs were serially numbered according to their frequency of occurrence in MEME bioinformatics tool.

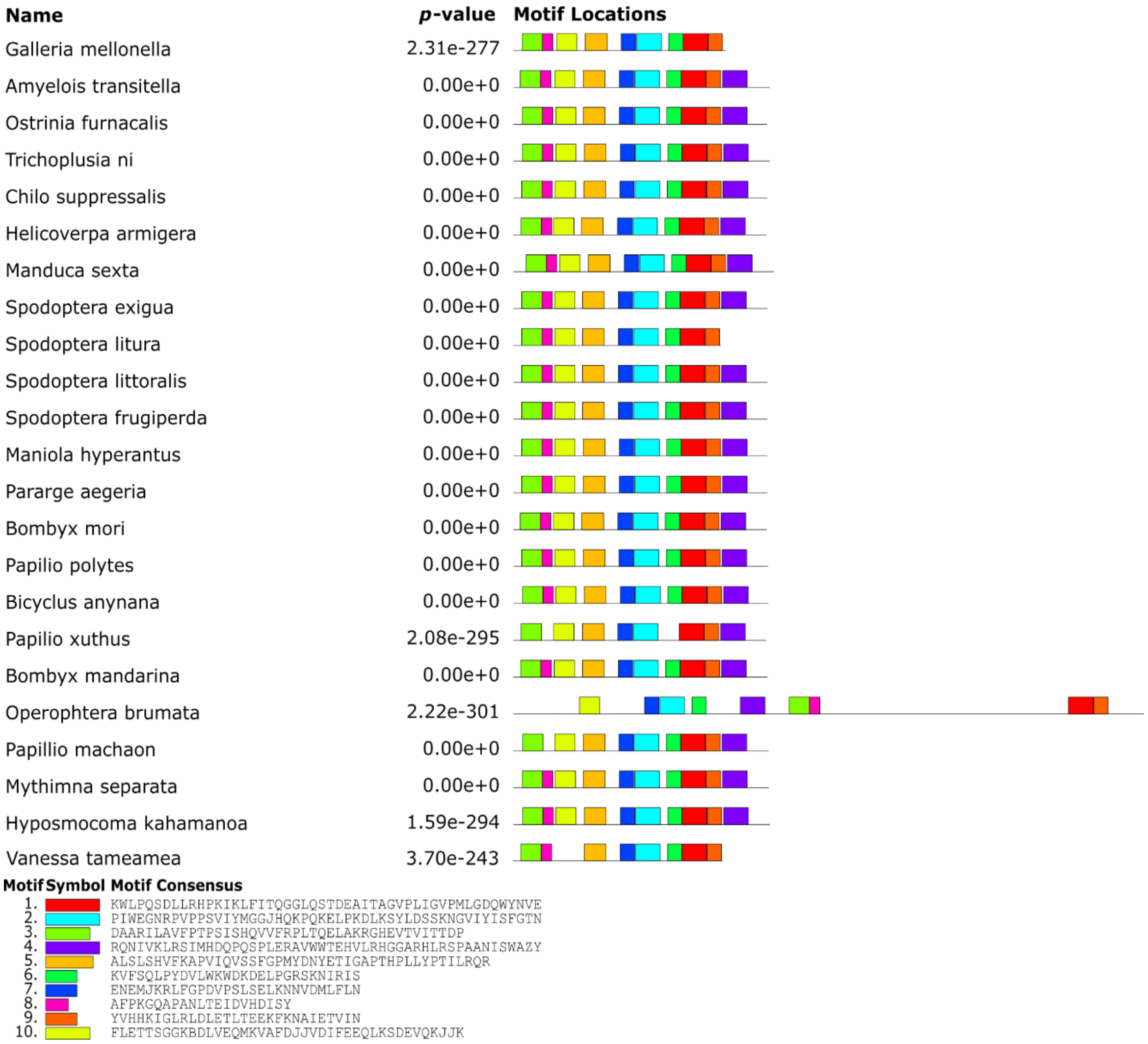

Supplementary Figure S11. The conserved motif distribution of α-amylase proteins across the different insect orders. Each categorized motif logo generated by MEME is displayed in differentially colored boxes. Legend (at the bottom right) depicts the protein sequence of corresponding motifs. Motifs were serially numbered according to their frequency of occurrence in MEME bioinformatics tool.

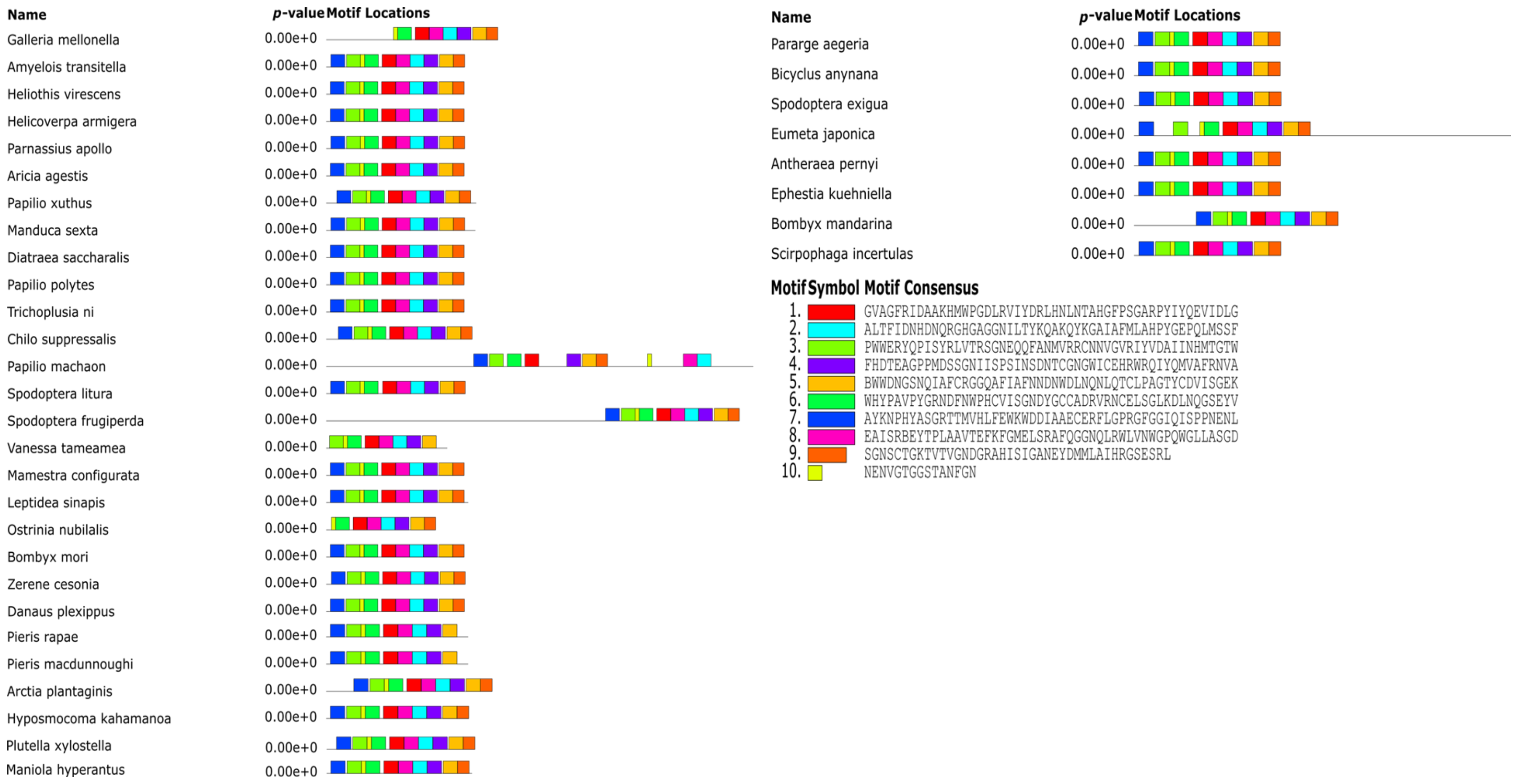

Supplementary Figure S12. The conserved motif distribution of arylphorin proteins across the different insect orders. Each categorized motif logo generated by MEME is displayed in differentially colored boxes. Legend (at the right hand side) depicts the protein sequence of corresponding motifs. Motifs were serially numbered according to their frequency of occurrence in MEME bioinformatics tool.

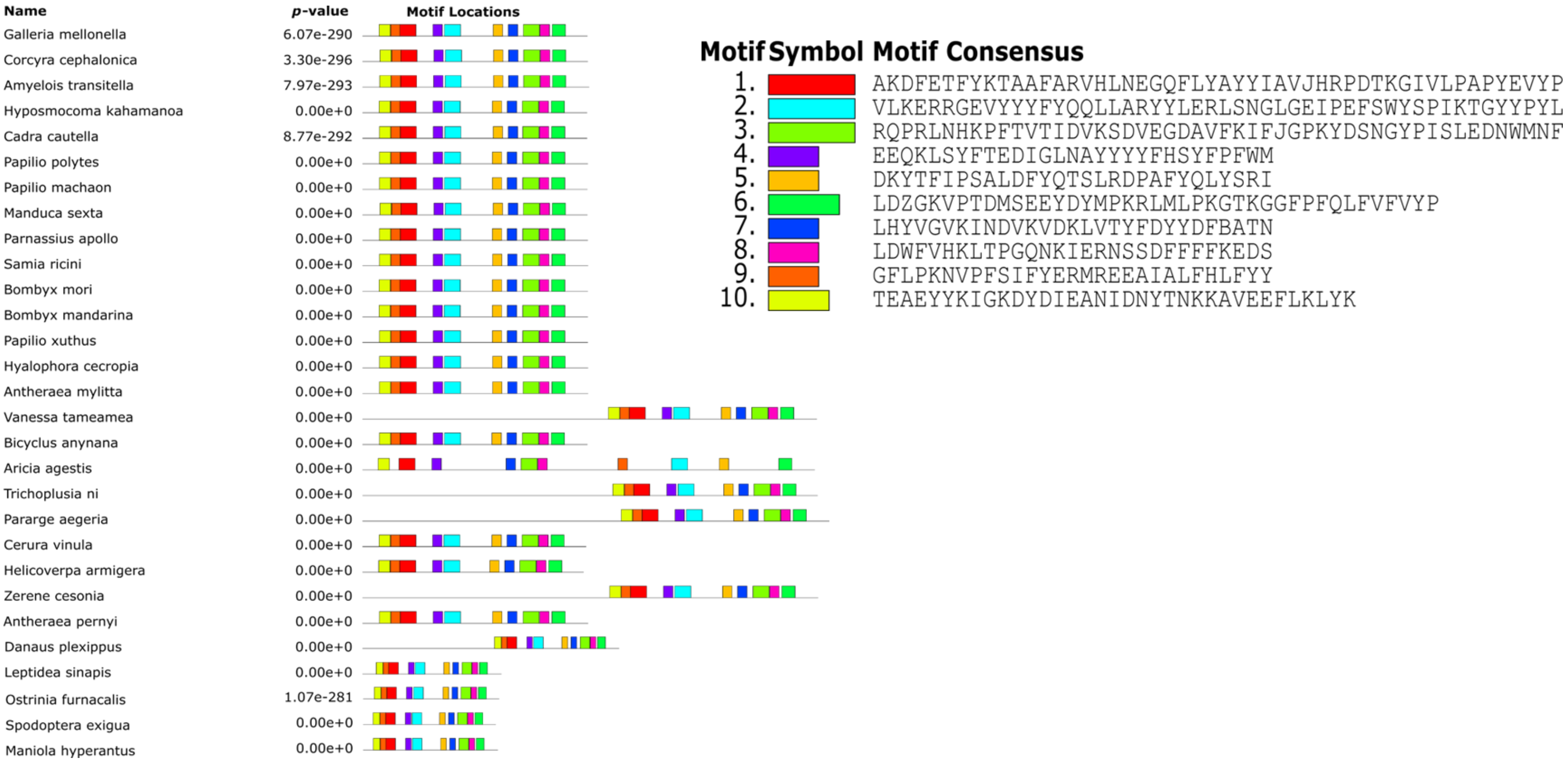

Supplementary Figure S13. The conserved motif distribution of GLTP proteins across the different insect orders. Each categorized motif logo generated by MEME is displayed in differentially colored boxes. Legend (at the bottom right) depicts the protein sequence of corresponding motifs. Motifs were serially numbered according to their frequency of occurrence in MEME bioinformatics tool.

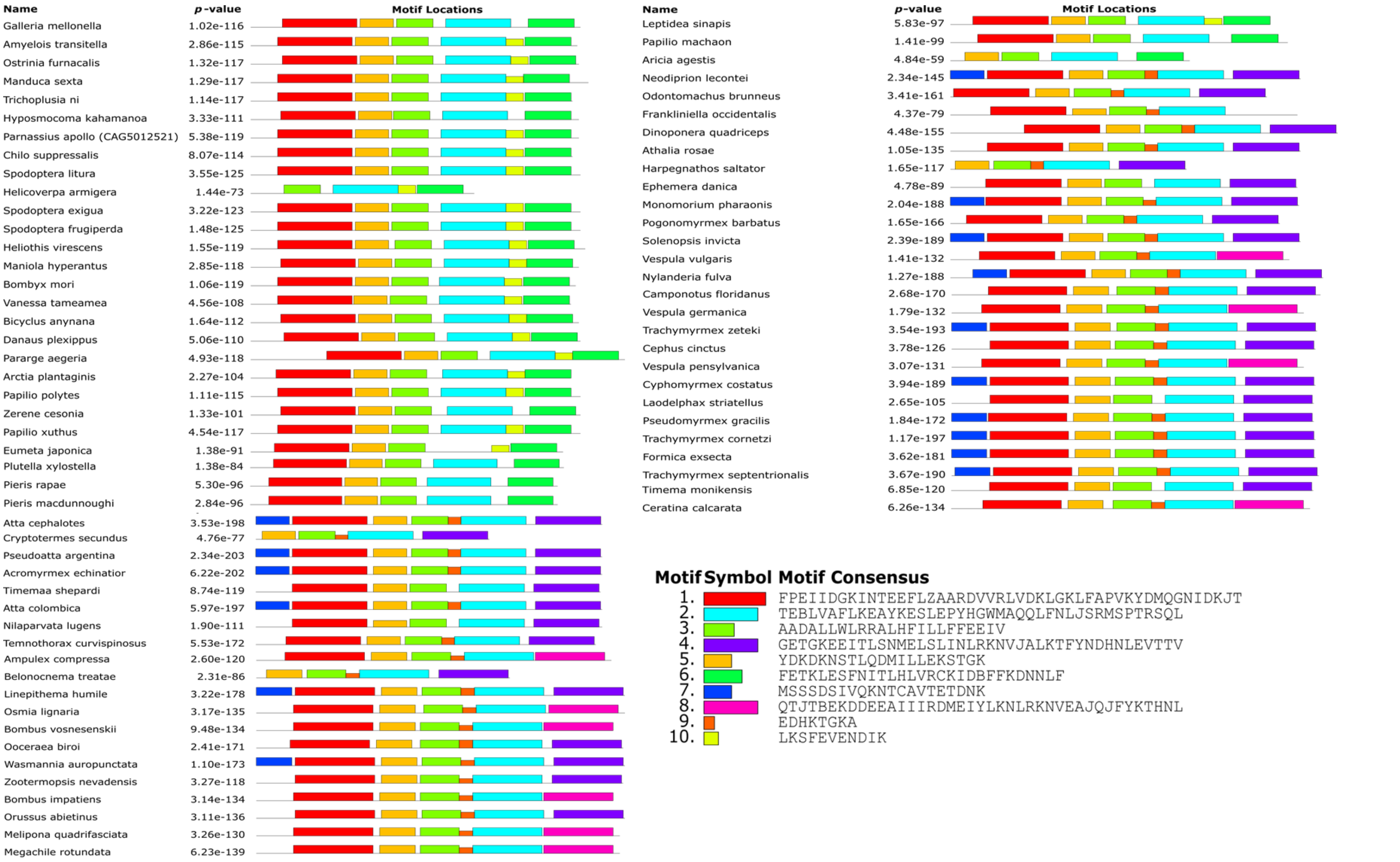

Supplement: Supplementary file 1 — Additionalfile 1: Supplementary Figure S1. Protein-protein interaction between Cry1AcF ligand and known gut receptors CAD, ABCC2, ALP and APN1. Cry1AcF bound with these receptors via a number of Pi interactions, hydrogen bonds and salt bridges. The ZDock scores (greater value indicates greater contact surface area between ligand and receptor) for Cry-CAD, Cry-ABCC2, Cry-ALP and Cry-APN1 complexes were 2321, 1907, 2189 and 2060 Å2, respectively. Domain I, II and III of Cry1AcF arehighlighted in magenta, ochre yellow and green color, respectively. Supplementary Figure S2. Evolutionary relationship of ADAM protein from G. mellonella with their corresponding homologues from other insect species. The phylogenetic treewas constructed in MEGA X software using Maximum Likelihood method; best model was selected via MODELTEST using Le and Gascuel method. Bootstrap consensus was inferred from 1000 replicates and branches corresponding to < 70% replicates were collapsed. The analyses included 61 amino acid sequences. NCBI accessionnumbers of different entries are provided in parentheses. All gaps and missingdata positions were eliminated after sequence alignment. Homo sapiens sequencefor the corresponding protein was used as the out-group (marked with ●), and G. mellonella entry is indicated in bold font. Entries in blue, black, red and green correspond to the members of the order Hymenoptera, Isoptera, Coleoptera and Lepidoptera, respectively. Supplementary Figure S3. Evolutionary relationship of prohibitin protein from G. mellonella with their corresponding homologues from other insect species. The phylogenetic tree was constructed in MEGA X software using Maximum Likelihood method; best model was selected via MODELTEST using Le and Gascuel method. Bootstrap consensus was inferred from 1000 replicates and branches corresponding to < 70% replicates were collapsed. The analyses included 88 amino acid sequences. NCBI accession numbers ofdifferent entries are provided in parenthe [file 12864_2022_8843_MOESM1_ESM.pdf]
