# Supplementary material for: RNAi-mediated knockdown of gut receptor-like genes prohibitin and α-amylase altered the susceptibility of Galleria mellonella to Cry1AcF toxin
Source: BMC Genomics. 2022 Aug 18;23:601. doi: 10.1186/s12864-022-08843-8 (PMC9389788; doi:10.1186/s12864-022-08843-8)
Supplement: Supplementary file 2 — Additional file 1: Supplementary Table S1. A summary of number of unigenes corresponding to different Cry toxin receptors from the NCBI non redundant database for G. mellonella transcripts/ESTs (E-value cut off < 1.0E-30). Supplementary Table S2. In silico Protein-Protein Interface analysis. Supplementary Table S3. Oligonucleotides used for RACE-PCR and verifying full-length cDNAsequence. Tm = 60°C. Supplementary Table S4. Oligonucleotides used for RNAi and RT-qPCR analysis. Tm = 60°C. [file 12864_2022_8843_MOESM2_ESM.pdf]

SUPPLEMENTARY TABLES

Title: **RNAi-mediated knockdown of gut receptors prohibitin and UDP-GT altered the susceptibility of *Galleria mellonella* to Cry1AcF toxin**

Authors: Tushar K. Dutta[1*], Abhishek Mandal[2], Artha Kundu[1], Victor Phani[3], Chetna Mathur[1], Arudhimath Veeresh[1], Rohini Sreevathsa[4]

Affiliation: [1]Division of Nematology, ICAR-Indian Agricultural Research Institute, New Delhi, 110012, India
[2]Division of Agricultural Chemicals, ICAR-Indian Agricultural Research Institute, New Delhi, 110012, India
[3]Department of Agricultural Entomology, College of Agriculture, Uttar Banga Krishi Viswavidyalaya, Dakshin Dinajpur, West Bengal, India
[4]ICAR-National Institute for Plant Biotechnology, New Delhi, 110012, India

Corresponding Author
[*]Dr. Tushar K Dutta
Division of Nematology
ICAR-Indian Agricultural Research Institute
New Delhi, India-110012
TEL: +91-11-2584-2721
Email: tushar.dutta@icar.gov.in; nemaiari@gmail.com

Supplementary Table S1. A summary of number of unigenes corresponding to different Cry toxin receptors from the NCBI non-redundant database for *G. mellonella* transcripts/ESTs (*E*-value cut off < $1.0E^{-30}$).

**Gene name**	**Number of unique transcripts**	**Reference sequence number and length (in parentheses) of the transcripts**
prohibitin	1	NW_022270177.1 (584 bp)
GLTP	1	NW_022272931.1 (1007 bp)
α-amylase	1	NW_022270666.1 (519 bp)
ADAM	1	NW_022266524.1 (1382 bp)
UDP-GT	5	NW_022277870.1 (815 bp), NW_022271647.1 (801 bp), NW_022276985.1 (786 bp), NW_022277371.1 (660 bp), NW_022267530.1 (234 bp)
arylphorin	49	NW_022275296.1 (783 bp), NW_022271163.1 (315 bp), NW_022273655.1, NW_022271732.1, NW_022271459.1, NW_022270048.1, NW_022269664.1 (301 bp), NW_022272446.1, NW_022272307.1 (310 bp), NW_022270367.1 (301 bp), NW_022269869.1 (305 bp), NW_022269199.1 (308 bp), NW_022268635.1 (309 bp), NW_022266129.1 (305 bp), NW_022277701.1, NW_022275502.1, NW_022274690.1, NW_022270926.1, NW_022270759.1, NW_022270666.1 (301 bp), NW_022270124.1 (305 bp), NW_022270008.1, NW_022269208.1, NW_022267677.1, NW_022267478.1, NW_022267349.1 (301 bp), NW_022266938.1 (311 bp), NW_022266898.1, NW_022266524.1 (301 bp), NW_022266217.1 (302 bp), NW_022266059.1 (301 bp), NW_022271951.1 (300 bp), NW_022278607.1 (309 bp), NW_022271782.1 (302 bp), NW_022268972.1 (305 bp), NW_022279190.1, NW_022277594.1, NW_022276972.1, NW_022276442.1, NW_022274893.1, NW_022272931.1 (301 bp), NW_022271647.1 (304 bp), NW_022269487.1, NW_022268286.1, NW_022266547.1, NW_022266300.1 (301 bp), NW_022266175.1, NW_022276401.1 (311 bp), NW_022275108.1 (306 bp)

Supplementary Table S2. *In silico* Protein-Protein Interface analysis.

Complex	Total Pi Interactions	Total Hydrogen Bonds	Total Salt Bridges	Ligand Contact Surface Area (Å²)	Ligand Polar Contact Surface Area (Å²)	Ligand Nonpolar Contact Surface Area (Å²)	Receptor Contact Surface Area (Å²)	Receptor Polar Contact Surface Area (Å²)	Receptor Nonpolar Contact Surface Area (Å²)	ZDock score (Å²)
Cry-prohibitin	13	7	0	943.22	429.17	514.05	1065.2	446.66	618.50	1510.855
Cry-GLTP	10	2	0	638.16	273.37	364.79	724.14	128.17	595.97	1961.207
Cry-α-amylase	9	2	0	912.55	418.69	493.86	920.61	325.43	595.18	2041.419
Cry-ADAM	8	13	1	853.42	390.03	463.39	993.41	494.34	499.07	1916.326
Cry-UDP-GT	5	10	0	945.53	493.16	452.36	976.29	422.23	554.06	1836.010
Cry-CAD	5	11	1	1028.73	540.13	488.60	1046.88	485.13	561.75	2321.610
Cry-ABCC2	9	2	0	912.55	418.69	493.86	920.61	325.43	595.18	1907.590
Cry-ALP	13	7	0	943.22	429.17	514.05	1065.2	446.66	618.50	2189.599
Cry-APN1	2	20	0	1496.50	769.87	726.63	1535.49	819.21	716.28	2060.400

Supplementary Table S3. Oligonucleotides used for RACE-PCR and verifying full-length cDNA sequence. T_m = 60°C

**Gene/primer detail**	**Orientation**	**Sequence (5'-3')**	**Purpose**
prohibitin cDNA	Sense	ATGGCTGCACAACTTTTCAACC	for obtaining first strand cDNA
	Antisense	ACTATGTTCTTTATAGGTGGAGTC	
prohibitin GSP1*	Antisense	GATGTGATTGATGGTAGTACTC	5'-RACE
prohibitin NGSP1**	Antisense	TGAACAGGATACGAAGGGTG	
prohibitin GSP2*	Sense	CAGGAAGCTGAAAAGGCAAG	3'-RACE
prohibitin NGSP2**	Sense	GCGCAAGCTGCCATTCTTC	
prohibitin cDNA	Sense	ATGGCTGCACAACTTTTCAACC	Full-length sequence verification
	Antisense	ACTATGTTCTTTATAGGTGGAGTC	
GLTP cDNA	Sense	CTAATGGCAACTCTAAATTTGACG	for obtaining first strand cDNA
	Antisense	ACTTTGCCTCGCAAGCACAC	
GLTP GSP1	Antisense	TGTGGTATTTTTTAACTGATCCTTC	5'-RACE
GLTP NGSP1	Antisense	GCTCTGTTTAGCCACAATATGC	
GLTP GSP2	Sense	CATGTGAAATAATCAATAAGGACTTG	3'-RACE
GLTP NGSP2	Sense	CTAAGAAAGTATTGCTACAAGAGG	
GLTP cDNA	Sense	TAGAATATAGGACTAACGGACAG	Full-length sequence verification
	Antisense	CGATGAATAAGGCATGTATCAC	
α-amylase cDNA	Sense	ATGACTGAGTTGCTACGATACC	for obtaining first strand cDNA
	Antisense	TTTAGACCTTACCTGTGGGC	
α-amylase GSP1	Antisense	CTACGTAGATCGTTGACAAGAC	5'-RACE
α-amylase NGSP1	Antisense	TGTGCACAAGTTCCTCCCG	
α-amylase GSP2	Sense	ATTCCAAGGTGGCAATCAGC	3'-RACE
α-amylase NGSP2	Sense	AACCACGATAACCAGAGAGG	
α-amylase cDNA	Sense	ATGACTGAGTTGCTACGATACC	Full-length sequence verification
	Antisense	TTTAGACCTTACCTGTGGGC	
ADAM cDNA	Sense	GGCCGCCATGTGCGTAGA	for obtaining first strand cDNA
	Antisense	CTGACGTATGAACTCACTTTCTA	
ADAM GSP1	Antisense	AGCCACAGTCCCTTCACACC	5'-RACE
ADAM NGSP1	Antisense	CAAGTTCAAGTTCAGTGACCGT	
ADAM GSP2	Sense	TGTAACGCCTGCAAAGAATATC	3'-RACE

ADAM NGSP2	Sense	GTTTTGTTTCAATATACCTAAGCTTC	
ADAM cDNA	Sense	CTAGCAATCGAAGGAGTAAGAG	Full-length sequence verification
	Antisense	AAAGTGCTATTCATCAGTAACTATTT	
UDP-GT cDNA	Sense	GATACATTTATTACCCTGTCTGTG	for obtaining first strand cDNA
	Antisense	TCCCATGTCTGACATACTGTTC	
UDP-GT GSP1	Antisense	TGACCTCGTTTAGCCAGTTC	5'-RACE
UDP-GT NGSP1	Antisense	CTATCGGAAGAATATAGTAATACAC	
UDP-GT GSP2	Sense	TGATGAAGAGAGTATTTGGTCC	3'-RACE
UDP-GT NGSP2	Sense	CCACCTAGCGTTGTCTATTTAG	
UDP-GT cDNA	Sense	CTAACTTCTAGCCGTGCTGTG	Full-length sequence verification
	Antisense	ATGTGCAATATACCTTTTGTCATTTA	
arylphorin cDNA	Sense	CTGTCCTCTTTTTAGCGGCG	for obtaining first strand cDNA
	Antisense	TAGGGCAGAGTCACAGGACG	
arylphorin GSP1	Antisense	TACGAATTCCAACCAATGTCTTC	5'-RACE
arylphorin NGSP1	Antisense	TTGCTCTGGAATAATCAGGCC	
arylphorin GSP2	Sense	AGAGTACTTGGAACCTTATTCTC	3'-RACE
arylphorin NGSP2	Sense	TTACTTGTCCGAGCAACAGC	
arylphorin cDNA	Sense	GAGAGGCACAGTTGGCTTC	Full-length sequence verification
	Antisense	TCTCTCTCCTCTTAGTGGTTAG	

*GSP, gene specific primer; **NGSP, nested gene specific primer.

Supplementary Table S4. Oligonucleotides used for RNAi and RT-qPCR analysis. T_m = 60°C

Primer/gene details	Orientation	Sequence (5'-3')	Purpose	PCR efficiency (%)	Standard curve R^2
prohibitin	Sense	GAGCTCCTTCTTTGTCCCTTGGGTGC [a]	dsRNA synthesis	NA	NA
	Antisense	AAGCTTTCAACAGCCTGCGTGAATTC [b]			
GLTP	Sense	GAGCTCCAAAACATATGCCCCAGTCAAA	dsRNA synthesis	NA	NA
	Antisense	AAGCTTTCTGGGGAAGTGTAGGTGAC			
α-amylase	Sense	GAGCTCTGGTCAATTGGGGTCCTCAA	dsRNA synthesis	NA	NA
	Antisense	AAGCTTGATTGCCACCGTTATCCCAC			
ADAM	Sense	GAGCTCGCCATCTTCAACGACCCATC	dsRNA synthesis	NA	NA
	Antisense	AAGCTTGCACATTGGTCATCTCCGTC			
UDP-GT	Sense	GAGCTCAACCGTCCTGTTCCACCTAG	dsRNA synthesis	NA	NA
	Antisense	AAGCTTCTTTTGGGTGCCTGAGAAGG			
arylphorin	Sense	GAGCTCTGAGCTCATACCAGTTGCCA	dsRNA synthesis	NA	NA
	Antisense	AAGCTTAGTTGGTAGAAGGCAGGGTC			
GFP	Sense	GAGCTCGCAGAGCGAGGTATGTAGGC [a]	dsRNA synthesis	NA	NA
	Antisense	AAGCTTCTGCCTCGGTGAGTTTTCTC [b]			
GLTP	Sense	CGCCCGTTATAGATGGAAAA	RT-qPCR	100.5	0.922
	Antisense	CCTCTACCAATCTCATCAAGCA			
prohibitin	Sense	TGCTCAATTTGATGCTGGAG	RT-qPCR	101.6	0.989
	Antisense	TTGCCTTTTCAGCTTCCTGT			
α-amylase	Sense	CATATGTGGCCTTCCGATCT	RT-qPCR	99.9	0.965
	Antisense	TTTGAACTCGGTGACAGCAG			
ADAM	Sense	CCCGTCATCTCCACCTCTTA	RT-qPCR	99.6	0.925
	Antisense	CTCGTAGGTTGCAGCACAAA			

UDP-GT	Sense	CCCGGATATTGGGCTATTTT	RT-qPCR	99.8	0.915
	Antisense	GCCGTTGAACACTCCTTCTC			
arylphorin	Sense	TGACCCTGCCTTCTACCAAC	RT-qPCR	100.2	0.944
	Antisense	AAGCTGTTGCTCGGACAAGT			
18S rRNA	Sense	CACATCCAAGGAAGGCAG	RT-qPCR	107.9	0.978
	Antisense	AGTGTACTCATTCCGATTACGA			
EF-1α	Sense	AACCTCCTTACAGTGAATCC	RT-qPCR	106.4	0.969
	Antisense	ATGTTATCTCCGTGCCAG			

[a] underlined sequence indicates *Sac*I endonuclease site.

[b] underlined sequence indicates *Hind*III endonuclease site.

NA, not applicable.
